# Dietary Cinnamaldehyde Enhances Growth Performance, Digestion, Immunity, and Lipid Metabolism in Juvenile Fat Greenling (*Hexagrammos otakii*)

**DOI:** 10.1155/2022/2132754

**Published:** 2022-11-02

**Authors:** Yixin Gu, Jian Han, Wenjie Wang, Yu Zhan, Huijie Wang, Wenyuan Hua, Yue Liu, Yafeng Guo, Zhuang Xue, Wei Wang

**Affiliations:** Key Laboratory of Applied Biology and Aquaculture of Northern Fishes in Liaoning Province, Dalian Ocean University, Dalian 116023, China

## Abstract

Fat greenling (*Hexagrammos otakii*) is a kind of economic fish that is widely consumed by human, and its intensive farming technology is making important progress. However, high-density farming may cause the occurrence of diseases in *H*. *otakii*. Cinnamaldehyde (CNE) is a new feed additive for aquatic animals and has a positive effect on disease resistance. In the study, dietary CNE was evaluated on the growth performance, digestion, immune response, and lipid metabolism of juvenile *H*. *otakii* (6.21 ± 0.19 g). Six experimental diets were formulated containing CNE at levels of 0, 200, 400, 600, 800, and 1000 mg/kg for 8 weeks. The percent weight gain (PWG), specific growth rate (SGR), survival (SR), and feeding rate (FR) were significantly increased by including CNE in fish diets regardless of the inclusion level (*P* < 0.05). The feed conversion ratio (FCR) was significantly decreased among the groups fed CNE supplemented diets (*P* < 0.05). A significant decrease in hepatosomatic index (HSI) was observed in fish fed 400 mg/kg-1000 mg/kg CNE compared to the control diet (*P* < 0.05). Fish-fed diets containing 400 mg/kg and 600 mg/kg CNE had a higher level of crude protein in muscles than the control diet (*P* < 0.05). Moreover, the activities of lipase (LPS) and pepsin (PEP) in the intestinal were markedly increased in juvenile *H. otakii*-fed dietary CNE (*P* < 0.05). Apparent digestibility coefficient (ADC) of dry matter, protein, and lipid was significantly increased with CNE supplement (*P* < 0.05). The activities of catalase (CAT) and acid phosphatase (ACP) in the liver were markedly enhanced by including CNE in juvenile *H*. *otakii* diets compared with the control (*P* < 0.05). The activities of superoxide dismutase (SOD) and alkaline phosphatase (AKP) in the liver were markedly enhanced in juvenile *H. otakii* treated with CNE supplements 400 mg/kg-1000 mg/kg (*P* < 0.05). Additionally, the levels of total protein (TP) in the serum were markedly increased by including CNE in juvenile *H*. *otakii* diets compared with the control (*P* < 0.05). In the CNE200, CNE400, and CNE600 groups, albumin (ALB) levels in the serum were markedly higher compared with that in the control (*P* < 0.05). In the CNE200 and CNE400 groups, the levels of immunoglobulin G (IgG) in the serum were significantly increased compared with that the control group (*P* < 0.05). The juvenile *H*. *otakii*-fed dietary CNE had lower triglycerides (TG) and total cholesterol (TCHO) levels in the serum than fish-fed CNE-free diets (*P* < 0.05). The gene expression of peroxisome proliferator-activated receptor alpha (*PPAR-α*), hormone-sensitive lipase (*HSL*), and carnitine O-palmitoyltransferase 1 (*CPT1*) in the liver was significantly increased by including CNE in fish diets regardless of the inclusion level (*P* < 0.05). However, fatty acid synthase (*FAS*), peroxisome proliferator-activated receptor gamma (*PPAR-γ*), and acetyl-CoA carboxylase alpha (*ACCα*) in the liver were markedly decreased with CNE supplements 400 mg/kg-1000 mg/kg (*P* < 0.05). The glucose-6-phosphate1-dehydrogenase (*G6PD*) gene expression levels in the liver were markedly decreased compared with the control (*P* < 0.05). The optimal supplementation level of CNE was shown by curve equation analysis to be 590.90 mg/kg.

## 1. Introduction

In the modern aquaculture industry, intensive aquaculture system has been widely promoted and applied in grass carp (*Ctenopharyngodon idella*), carp (*Cyprinus carpio*), tilapia (*Oreochromis niloticus*), channel catfish (*Ictalurus punctatus*), and other fishes, which can create greater economic value for farmers [1–4]. On the other hand, farming of fish under intensive culture system with high densities can trigger a great risk of stressful conditions, which would suppress the immune system and make fish more prone to the diseases resulting in extremely mortalities and significant economic losses [5]. In recent years, a variety of antibiotics, such as flavomycin, bacitracin zinc, salinomycin, and enramycin, have been used to alleviate the pressure of various infectious diseases in farmed fish [6, 7]. However, the long-term use of antibiotics can lead to drug resistance and drug residues in the body of fish [8]. Moreover, beneficial microorganisms such as *Lactobacillus* and *Vibrio* in the gut of aquatic animals play a key role in stabilizing metabolic balance. Antibiotics can inhibit the production of beneficial microorganisms, which leads to an imbalance in the intestinal microecology and causes intestinal inflammation, intestinal mucosal shedding, and other diseases [9]. Therefore, seeking new feed additives to replace antibiotics has become the focus of fish researchers and nutritionists.

Phytonutrients are considered to be multifunctional and antibiotic-free feed additives, which are beneficial to health in the diets [10]. Cinnamon (*Cinnamomum zeylanicum*) is one of the phytonutrient species and includes compounds such as polysaccharides, polyphenols, and flavonoids, which are widely used in medicinal materials and the food industry [11]. Cinnamaldehyde (CNE) is the main component of cinnamon, which is an aromatic aldehyde organic compound with the activity of antibacterial, antifungal, anticancer, and antifibrotic [12, 13]. CNE can be used as a flavoring agent in drinks and as an additive in diets [14]. In the field of nutrition research, CNE has been used as a feed additive in the diets of terrestrial animals and birds, and a certain dose of CNE can promote their growth performance and improve disease resistance and antibacterial ability [15–17]. In recent years, CNE has been used in the diet of aquatic animals. The study suggests that CNE at 1000 mg/kg was able to boost the percent weight gain, specific growth rate, and protein efficiency in tongue sole (*Cynoglossus semilaevis*) [18]. Amer et al. [19] have demonstrated that adding 2 mL/kg CNE to the diet of Nile tilapia (*Oreochromis niloticus*) can enhance antioxidant activity and immune status. Bandeira Junior et al. [20] pointed out that supplementation of 1 mL/kg cinnamon essential oil increased length and weight gain for 60 days in silver catfish (*Rhamdia quelen*) and increased the activity of superoxide dismutase in the liver by reducing levels of thiobarbituric acid reactive species.

Fat greenling (*Hexagrammos otakii*) belongs to Scorpaeniformes, which is a marine carnivorous fish. *H*. *otakii* is mainly distributed in the Korean Peninsula, Japan, China, and Russia. *H*. *otakii* is an important commercial variety due to its excellent meat quality, rich protein, and nutrition [21]. However, due to higher price and increasing demand of the farming of *H. otakii*, fish meal (FM) is compelled to search alternative protein sources. Chicken gut meal (CGM) is reasonably priced and nutritious, which can partially or fully replace FM in fish [22, 23]. One of our previous studies showed that replacing FM with 75% CGM in the diet of juvenile *H*. *otakii* caused abnormal lipid metabolism (unpublished research). Thence, we need to treat lipid metabolism by improving dietary formula in fish. Since CNE inhibits the release of adrenaline and adrenocorticotropic hormone (ATCH) to fatty acid and facilitates the fat synthesis of glucose in vertebrates, it can be applied as a replacement of insulin to prevent diabetes [24]. Based on nontargeted metabolomics, CNE has been shown to ameliorate disturbances of glucose and lipid metabolism in mice by activating AMP-activated protein kinase (AMPK) [25]. Moreover, according to market research analysis, the price of CNE (￥ 3.2 yuan/kg) and CGM (￥ 7 yuan/kg) is lower than that of FM (￥ 13.1 yuan/kg). Therefore, the combined use of CGM and CNE in juvenile *H. otakii* diets can save cost and may improve lipid metabolism, which is of great importance for the realization of intensive breeding of *H. otakii* meaning. To the best of our knowledge, this study is the first to evaluate the effects of dietary CNE supplementation on growth performance, digestion, immune parameters, and relative gene expression of lipid metabolism in juvenile *H. otakii* and also provided a theoretical reference for the development and utilization of new feed additive in the *H*. *otakii* diet.

## 2. Materials and Methods

### 2.1. Experimental Diets and Design

The formulation and proximate composition of the experimental diets are listed in Table 1. FM and CGM were the major protein source; fish oil was the main fat source. Six experimental diets were formulated with CNE at 0 (CNE0), 200 mg/kg (CNE200), 400 mg/kg (CNE400), 600 mg/kg (CNE600), 800 mg/kg (CNE800), and 1000 mg/kg (CNE1000) diet. All of the dry ingredients were finely ground into powder through 60 mesh screens, distilled water was added to mix with them, and the 2 mm pellet feed was made by the granulator, oven-dried at 43°C for approximately 24 h, sealed in polythene bags, and stored at -20°C until used.

### 2.2. Experimental Methods

#### 2.2.1. Experiment Feeding Management

Juvenile *H*. *otakii* was obtained from the key laboratory of applied biology and aquaculture of fish (Dalian, China). Select healthy, disease-free 270 juvenile fishes (average initial weight (6.21 ± 0.19) g was randomly assigned to 18 (30 cm × 75 cm) cages in the circulating pool (predisinfection)). Each diet was randomly assigned to three replicate groups of fish. The experimental fish were been acclimated with the experimental diet for one week before the experiment. Feeding was performed twice a day (9:00 and 16:00) and under a natural photoperiod during the 8-week feeding trial. The water temperature was 10 ± 2°C, salinity was 26-30, pH was 7.8 ± 0.4, dissolved oxygen was 6.6 ± 0.7 mg/L, and ammonia nitrogen content < 0.1 mg/L.

#### 2.2.2. Sample Collection and Analysis

After the feeding experiment, the fish was starved for 24 h prior to sampling. Then, the 10 juvenile *H. otakii* in each cage were randomly selected and calculated the percent weight gain (PWG), specific growth rate (SGR), feed conversion ratio (FCR), condition factor (CF), and feeding rate (FR). Juvenile *H*. *otakii* were anesthetized with 100 mg/L tricalcium methanesulfonate (MS-222) and subjected to vivisection. Blood was collected from the base of the caudal-fin then centrifuged at a rate of 7000 rpm/min for 10 min at 4°C, and the supernatant was collected to determine the serum biochemical indexes. Superoxide dismutase (SOD), catalase (CAT), malondialdehyde (MDA), acid phosphatase (ACP), alkaline phosphatase (AKP), aspartate aminotransferase (AST), and alanine aminotransferase (ALT) were determined from the liver. Fish viscera and whole intestines were collected and measured for lipase (LPS), amylase (AMS), pepsin (PEP), hepatosomatic index (HSI), viscersomatic index (VSI), and intestosomatic index (ISI). Feces of fish were collected by siphon method [26] to determine apparent digestibility coefficient (ADC). Lastly, the muscle of the fish was preserved -20°C to measure the proximate nutritional composition of the muscles.

#### 2.2.3. Growth Performance

The growth performance was evaluated using the following parameters.

(1) $$\begin{array}{c} \text{Percent weight gain }\left(\text{PWG},\%\right)=\frac{\left(\text{final weight}-\text{initial weight}\right)}{\text{initial weight}}\times 100,\\ \text{Specific growth rate }\left(\text{SGR},\%\text{body weight}/\text{day}\right)=\frac{\left[\ln\left(\text{final weight}\right)–\ln\left(\text{initial weight}\right)\right]}{\text{feeding trial days}}\times 100,\\ \text{Feed conversion ratio }\left(\text{FCR}\right)=\frac{\text{total feed consumption }}{\text{wet weight gain}},\\ \text{Hepatosomatic index }\left(\text{HSI},\%\right)=\frac{\text{liver wet weight}}{\text{body wet weight}}\times 100,\\ \text{Viscerosomatic index }\left(\text{VSI},\%\right)=\frac{\text{visceral wet weight}}{\text{body wet weight}}\times 100,\\ \text{Intestosomatic index }\left(\text{ISI},\%\right)=\frac{\text{intestine wet weight}}{\text{body wet weight}}\times 100,\\ \text{Condition factor }\left(\text{CF},\text{g}/{\text{cm}}^{3}\right)=\frac{\text{body weight}}{\text{body }{\text{length}}^{3}}\times 100,\\ \text{Survival }\left(\text{SR},\%\right)=\frac{\left(\text{last amount of fish}\right)}{\left(\text{initial amount of fish}\right)}\times 100,\\ \text{Feeding rate }\left(\text{FR},\%/\text{d}\right)=\frac{\text{feed intake in dry matter}}{\left[\left(\text{initial body weight}+\text{final body weight}\right)/2\right]/\text{feeding trial days }}\times 100. \end{array}$$

#### 2.2.4. Proximate Composition in Muscles and Diet Analysis

According to the standard method, the approximate composition of diets and muscles was analyzed [27]. Moisture was determined by oven drying at 105°C to constant weight, and ash passed through a muffle furnace at 550°C for 5 h. Crude protein (*N* × 6.25) was digested with acid and analyzed by the Kjeldahl method. Crude lipid was determined by the petroleum ether extraction.

#### 2.2.5. Digestibility Trial

To calculate apparent digestibility coefficients (ADCs) of the experimental diets, a quantity of 0.2% of Cr_2_O_3_ was used in each test diet as an inert marker for estimation of apparent digestibility coefficients. According to the standard method, the approximate composition of feces was analyzed [27]. The determination Cr_2_O_3_ in diets and feces refers to Zheng et al. [28], calculated as follows:

(2) $$\begin{array}{c} {\text{ADC}}_{\left(\text{dry matter}\right)}\left(\%\right)=100\times \left(\frac{1-{\text{Cr}}_{2}{\text{O}}_{3}\text{ in the diet}}{{\text{Cr}}_{2}{\text{O}}_{3}\text{ in the feces}}\right),\\ {\text{ADC}}_{\left(\text{nutrient}\right)}\left(\%\right)=100\times \left[1-\left(\frac{\text{nutrient content in the feces}}{\text{nutrient content in the diet}}\right)\times \left(\frac{{\text{Cr}}_{2}{\text{O}}_{3}\text{ in the diet}}{{\text{Cr}}_{2}{\text{O}}_{3}\text{ in the feces}}\right)\right]. \end{array}$$

#### 2.2.6. Intestinal, Liver, and Serum Biochemical Parameter Measurement

The intestinal digestive enzymes included lipase (LPS) (U/g prot), amylase (AMS, U/mg prot), and pepsin (PEP, U/mg prot) (acid pepsin, pH: 1.5-5); the liver immune and metabolic enzymes included superoxide dismutase (SOD, U/mg prot), catalase (CAT, U/mg prot), malondialdehyde (MDA, nmol/g prot), acid phosphatase (ACP, U/g prot), alkaline phosphatase (AKP, U/g prot), aspartate aminotransferase (AST, U/g prot); and alanine aminotransferase (ALT, U/g prot); the serum immune and metabolic enzymes included total protein (TP, g/L), immunoglobulin G (IgG, g/L), albumin (ALB, g/L), triglycerides (TG, mmol/L), total cholesterol (TCHO, mmol/L), aspartate aminotransferase (AST, U/L), and alanine aminotransferase (ALT, U/L) by using lipase assay kit (colorimetry), amylase assay kit (starch iodine colorimetry), pepsin assay kit (colorimetry), superoxide dismutase assay kit (hydroxy-amine method), catalase assay kit (ammonium molybdate method), malondialdehyde assay kit (TAB method), alkaline phosphatase assay kit (visible light colorimetry), acid phosphatase assay kit (colorimetry), total protein assay kit (Coomassie brilliant blue method), albumin assay kit (colorimetry), immunoglobulin G assay kit (immunoturbidimetry), triglycerides assay kit (colorimetry), total cholesterol assay kit (colorimetry), aspartate aminotransferase assay kit (colorimetry), and alanine aminotransferase assay kit (colorimetry). All the kits were provided by the Jiancheng Bioengineering Institute (Nanjing, China) (http://www.njjcbio.com/).

#### 2.2.7. Quantitative Real-Time PCR Analysis

RNA was extracted from the liver of juvenile *H*. *otakii* by using the Trizol method [29]. Ultra-microphotometer (Biochrom Technologies, UK) was used to assess the quantity and quality of total RNA. The 260/280 nm absorbance ratios of all selected samples were ranged from 1.85 to 2.00. Total RNA was used as a template to synthesize cDNA for preservation at -20°C, according to the reverse transcription kit provided by Baisai Biotechnology Co. (Shanghai, China). The primer sequences used are shown in Table 2. *β*-Actin gene was used as a housekeeping gene. The fluorescence quantitative PCR reaction system was 20 *μ*L: 0.6 *μ*L upstream primer, 0.6 *μ*L downstream primer, 10 *μ*L 2× Talent qPCR PreMix, 1 *μ*L cDNA, and 7.8 *μ*L RNase-Free ddH_2_O. Quantitative real-time PCR (qRT-PCR) analysis was performed in a quantitative thermal cycler (Roche, Light cycler 96, Basel, Switzerland). The cycling conditions of qRT-PCR were used as follows: 95°C for 3 min, 40 cycles for annealing at 60°C for 15 s, and denaturation at 95°C for 5 s. Temperature was increased from 55°C to 95°C to conduct melting curve analysis. Agarose gel electrophoresis of the final product was conducted which confirmed the presence of single amplicons. Standard curves were generated using six different dilutions (in triplicate). The data of expression analysis was analyzed by using the 2^−ΔΔCT^ method [30].

#### 2.2.8. Statistical Analysis

**Figure 1:**
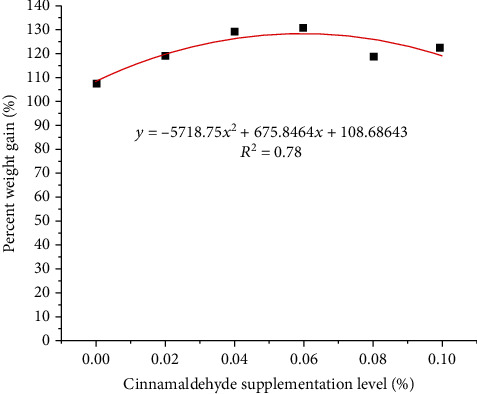
Curve chart about the relationship between the cinnamaldehyde supplementation level (%, *X*) and percent weight gain (%, *Y*) in juvenile *H*. *otakii* for 8 weeks.

The experiment data were analyzed using one-way ANOVA with the software SPSS 19.0 (SPSS, Chicago, Illinois). Data were represented as mean ± standard error of mean (SEM). Prior to statistical analyses, raw data were diagnosed for normality of distribution and homogeneity of variance with the Kolmogorov-Smirnov test and Levene test, respectively. Mathematical transformations were applied if at least one of the assumptions was not verified. Each treatment group was compared with the control group. To adjust for multiple comparisons among the groups, Duncan's method was used, and significant difference was set at *P* < 0.05. A curve equation analysis was conducted to analyze in response to dietary CNE of juvenile *H*. *otakii* (Figure 1).

## 3. Results

### 3.1. Growth Performances

CNE had a significant positive effect on the growth performance parameters of juvenile *H*. *otakii* (Table 3). The best PWG, SGR, FCR, and FR were observed in the CNE400 and CNE600 groups (*P* < 0.05). VSI, ISI, and CF have no significant differences among dietary groups (*P* > 0.05). HSI was significantly decreased in the CNE400, CNE600, CNE800, and CNE1000 groups compared to the control group (*P* < 0.05). Moreover, the groups of supplementation CNE improved SR compared to the control group (*P* < 0.05).

A curve analysis was used to estimate the optimal supplementation level of CNE. Based on PWG, curve equations were *y* = −5718.75*x*2 + 675.8464*x* + 108.68643 (*R*^2^ = 0.78). The vertices of the lower axis of the curve were at 0.059090%.

### 3.2. Muscle Compositions

The muscle compositions of juvenile *H*. *otakii* are shown in Table 4. Moisture, crude lipid and ash were not significantly difference among treatments (*P* > 0.05). Meanwhile, crude protein was significantly increased in fish groups of CNE400 and CNE600 compared with the control group (*P* < 0.05).

### 3.3. Digestive Enzyme Parameters

The digestive enzyme parameters of juvenile *H*. *otakii* after an 8-week experimental period are shown in Table 5. Supplementation CNE groups increased the activities of LPS and PEP (*P* < 0.05). However, there was no difference in intestinal AMS activity among the groups (*P* > 0.05).

### 3.4. Apparent Digestibility Coefficient Parameters

The apparent digestibility coefficient parameters of juvenile *H*. *otakii* after an 8-week experimental period are shown in Table 6. The digestibility coefficient of protein, lipid, and dry matter was significantly increased compared with the control group (*P* < 0.05).

### 3.5. Liver Biochemical Parameters

Liver biochemical parameters are presented in Table 7. There was no significant difference in MDA, AST, and ALT among all treatments (*P* > 0.05). Moreover, the groups of CNE400, CNE600, CNE800, and CNE1000 showed significantly increased SOD and AKP activities compared with the control group (*P* < 0.05). Additionally, higher significant CAT and ACP activities were observed in fish groups received diets supplemented with CNE in comparison with the control group (*P* < 0.05).

### 3.6. Serum Biochemical Parameters

The serum biochemical parameters of juvenile *H*. *otakii* are presented in Table 8. No significant differences were detected in serum AST and ALT among different experimental groups (*P* > 0.05). However, IgG was significantly increased in CNE200 and CNE400 groups compared to the control group (*P* < 0.05). CNE supplemented fish showed higher significant levels of TP unlike the control group (*P* < 0.05). ALB level was significantly increased in CNE200, CNE400, and CNE600 groups (*P* < 0.05). Additionally, the levels of TG and TCHO were significantly reduced with CNE supplementation (*P* < 0.05).

### 3.7. Expression of Genes Involved in Lipid Metabolism

**Figure 2:**
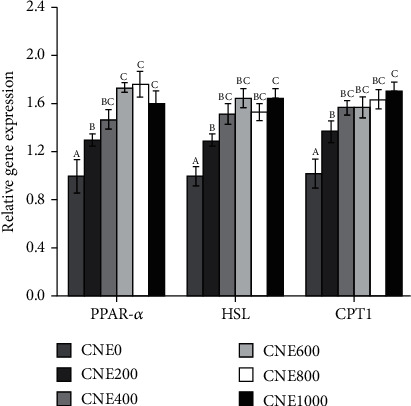
Relative gene expression of *PPAR-α*, *HSL*, and *CPT1* in juvenile *H*. *otakii*-fed diets supplemented with CNE levels. Mean values for the same gene with different letters were significantly different (*P* < 0.05).

**Figure 3:**
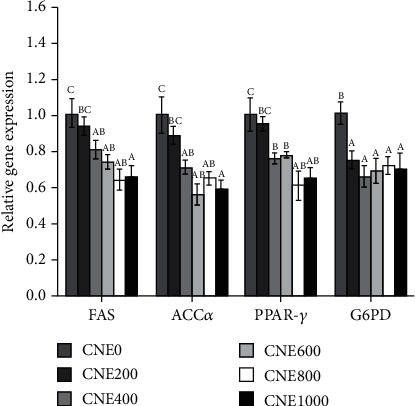
Relative gene expression of *FAS*, *ACCα*, *PPAR-γ*, and *G6PD* in juvenile *H*. *otakii*-fed diets supplemented with CNE levels. Mean values for the same gene with different letters were significantly different (*P* < 0.05).

The relative gene expression involved in lipid metabolism was presented in Figures 2 and 3. Fish-fed diets in groups of CNE400, CNE600, CNE800, and CNE1000 had lower expression of *FAS*, *ACCα*, and *PPAR-γ* than those fed the control diet group (*P* < 0.05). The relative gene expression of *G6PD* was significantly decreased with CNE supplemented (*P* < 0.05). Moreover, the groups of supplementations CNE increased gene expression of *CPT1*, *PPAR-α*, and *HSL* as compared to the control group (*P* < 0.05).

## 4. Discussion

In high-density farming, several phytonutrients have been shown to increase profits by enhancing fish growth and immunity [31]. Growth performance can directly reflect the response of fish to diet. As a cold-water fish, *H*. *otakii*'s growth rate and metabolism are much slower than those of warm- and hot-water fish. Therefore, CNE is usually added to fish feed as a growth promoter. There are two reasons for the addition of CNE to the diet to improve fish growth performance. On the one hand, feed intake is one of the factors affecting the growth performance of fish. Appetite enhancement has been documented to be a potential mechanism for the increase in feed intake [32–34]. Plant essential oils are aromatic and volatile compounds that act predators in fish diets. As a kind of plant essential oil, CNE can be used as a food attractant for fish to enhance feed intake [35]. This study indicated that the feeding rate was boosted with CNE supplement in the diets, which is a reason that improved growth performance of juvenile *H*. *otakii*. Abd El-Hamid et al. [36] indicated that dietary supplementation of CNE improved feed intake and specific growth rate of Nile tilapia. Supplementation of 0.75% cinnamon leaves in the diet enhanced the growth performance and substrate utilization of carp and yellow catfish (*Pelteobagrus fulvidraco*) [37, 38]. Similar results were also found in pigs, cattle, and growing lambs [39–41]. On the other hand, there is a close relationship between fish digestion and absorption of diet and growth performance [42]. In the present study, the digestive enzymes and apparent digestibility coefficient of fish were improved with CNE supplement. CNE can promote the digestion and absorption of nutrients and improve growth performance by inhibiting intestinal bacteria such as *Escherichia coli* (Gram-negative), *Salmonella* (Gram-negative), and *Shigella* (Gram-negative) [43, 44]. Previous studies have shown that CNE can boost growth performance by regulating the activity of broiler chicken gut microbiota [45]. Zhou et al. [46] suggested that dietary supplementation with CNE increased the activities of trypsin, amylase, lipase, sodium-potassium-ATPase, and intestinal creatine kinase, which in turn enhanced grass carp's growth performance. In the present study, the percent weight gain and specific growth rate of fish were enhanced following dietary CNE supplementation. These results stem from the fact that the addition of CNE to the diet improved the feed intake and digestibility of fish, which boosted growth performance of fish. Based on curve analysis, the optimal supplementation CNE level is recommended to be 590.90 mg/kg for juvenile *H*. *otakii*.

Among fish body indices, CF, HSI, VSI, and ISI reflect the nutritional and physiological status of fish. The results of the present study showed that CF, VSI, and ISI did not change with supplementation of CNE in the diet during the treatment. However, HSI was significantly decreased in the CNE400, CNE600, CNE800, and CNE1000 groups, which was consistent with previous studies performed on striped catfish (*Pangasianodon hypophthalmus*) [47]. A reduction in the percentage of HSI indicated that dietary CNE supplementation reduces lipid and glycogen content in the liver. CNE maintains insulin hormones by lowering blood sugar and lipid levels, and maintaining healthy liver activity [48].

Muscle nutrient composition may reflect fish response to dietary macronutrients. Fish tissue is composed of many primitive myoblasts, which fuse into multinucleated myotubes and develop into mature muscle tissue [49]. In the present study, moisture, crude lipid, and ash in muscle were not affected by supplementation CNE of diet. However, crude protein was higher in fish groups of CNE400 and CNE600. The amount of muscle protein is closely related to growth. Flavonoids are abundant in cinnamon, which are reported to show a positive effect on muscle quality and muscles cell differentiation as feed additives [50]. Xu et al. [51] have demonstrated that addition of 0.06% lotus leaf flavonoids could regulate muscle growth by improving the mRNA expression of fibroblast growth factor 6 b (FGF6b) in grass carp. Villasante et al. [52] showed that anthocyanin mixture (120 *μ*M of peonidin chloride, 50 *μ*M of cyanidin chloride, and 40 *μ*M of pelargonidin chloride) could boost cell survival in fish muscle cells by inducing gene expression pattern in accordance with a delay toward terminal differentiation. Therefore, the increase in muscle crude protein in this study was attributed to the improvement of fish muscle cell differentiation by flavonoids in cinnamon.

The antioxidant defenses and nonspecific immune systems in fish are highly linked to their health and immune mechanisms. SOD, CAT, and MAD prevent damage by reactive oxygen species and maintain a balance between free radical production and scavenging [53]. The present study described that the activities of SOD and CAT were increased with CNE supplementation. Some common phytonutrients such as soybean isoflavones and curcumin have major antioxidant action [33, 54]. Studies have shown that phytonutrients can scavenge free radicals through single-electron transfer antioxidant capacity [55]. Moreover, several studies indicated that dietary CNE boosted meat quality by increasing antioxidant capacity in animals, thereby protecting the product from spoilage [56, 57]. The nonspecific immune system is vital for fish, acting as the primary line of protection and driving force of adaptive immunity [58]. The study indicated that CNE as dietary additives trigger toll-like receptors to induce proinflammatory and chemokines, which drive the activation of innate immunity [59]. Faikoh et al. [60] found that CNE enhanced nonspecific immunity and survival in zebrafish (*Danio rerio*) under challenge with *Vibrio vulnificus* or *Streptococcus agalactiae*. Shan *et al*. [61] also pointed out that yesso scallop (*Patinopecten yessoensis*) treated with CNE enhanced the activities of ACP and AKP to prevent *Vibrio* infection. Our work found a significant stimulation in nonspecific immunity, as indicated by the increase in activities of ACP and AKP in fish due to dietary supplementation of CNE.

Serum biochemical markers are considered important to measure overall health of fish and are affected by animal developmental stages and nutritional levels. In this study, the levels of TP, ALB, and IgG were increased in fish fed CNE [62]. TP, ALB, and IgG are indicators for assessing various immunity in the diet, and the increase in their levels is attributed to the improvement of nonspecific immunity. However, TG and TCHO of fish were decreased following dietary supplementation with 200 mg/kg CNE. TG and TCHO are crucial components of animal fat, which are involved in the transport of various lipoprotein particles in plasma. TG and TCHO accumulation in serum could be explained by lipid transport impediment [63]. This study shows that dietary CNE supplementation can improve the fat metabolism status of fish. AST and ALT are one of the most crucial indicators to evaluate the health of liver metabolism. Abnormal protein metabolism can be explained by AST and ALT in the liver entering the blood [64]. In the present study, we notice that dietary CNE did not have a favorable effect on the hepatic protein metabolism status of the juvenile *H*. *otakii*. On the other hand, studies showed that CNE could restore the activities of AST and ALT in damaged liver tissue and downregulate the expression of interleukin-6 (IL-6), interleukin-1*β* (IL-1*β*), cyclooxygenase-2 (Cox-2), and tumor necrosis factor receptor type-2 (TNFR-2) inflammation-related protein and genes [65]. In addition to difference in species, environmental factors, as well as different metabolic cycles, could be the main reasons for differences in protein metabolism.

Lipids are composed of fatty acids and proteins, which are the main organic constituents of teleost fish. Fatty acids play an important role as a source of metabolic energy in fish growth [66]. However, the abnormal lipid metabolism in fish is a risk factor for diabetes, hyperlipidemia, and metabolic diseases [67, 68]. In recent years, several studies have demonstrated the positive role of CNE in the treatment of lipid metabolism disorders. Kaur et al. [69] have investigated the ability of cinnamon to reduce obesity-related metabolic disturbances in a zebrafish by means of blood glucose levels, serum triglyceride analysis, and Oil Red O staining. Setiawati et al. [70] pointed out that cinnamon leaf extract and powder more effectively increased high density lipoprotein levels of Asian catfish (*Clarias batrachus*) and reduced fat in the liver. However, research on lipid metabolism gene expression and CNE is still in its infancy.

Lipid homoeostasis is maintained in fish through a balance of catabolic and anabolic processes [71]. Fatty acids are catabolized in mitochondria or peroxisomes via the *β*-oxidation pathway [72]. Peroxisome proliferator-activated receptor (PPAR) binds to peroxisome proliferator response elements in the promoter reigns of target genes, which are involved in *β*-oxidation, such as *PPAR-α*, *CPT1*, and *HSL*. These genes are involved in the intracellular transport of fatty acids destined of catabolism [73]. As a subtype of *PPAR*, *PPAR-α* induces the intensity of *β*-oxidation by regulating *CPT1*. Fang et al. [74] pointed out that the expression of *PPAR-α* and *CPT1* was significantly increased by fat deposition in pompano (*Trachinotus ovatus*). As a downstream gene of *PPAR-α*, *HSL* can hydrolyze triglycerides, diglycerides, and monoglycerides [75]. Fish steatohepatitis is accompanied by a significant downregulation of *HSL* expression. A small amount of *HSL* is not sufficient to adequately catalyze the release of fatty acids from TG [76].

Conversely, fatty acids can be synthesized de novo by pathways that are activated by sterol regulatory element-binding protein (Srebp). Srebp have many target genes with examples of those in lipid metabolism including *FAS*, *PPAR-γ*, *ACCα*, and *G6PD* [77]. FAS is a multifunctional enzyme that uses acetyl-CoA as a primer, malonyl-CoA as a two-carbon donor, and nicotinamide adenine dinucleotide phosphate (NADPH) produced by *G6PD* as a reducing agent to catalyze long-chain fatty acid synthesis [78]. *ACCα* can be used for the ATP-dependent carboxylation of acetyl-CoA to generate malonyl-CoA, which is involved in the regulation of vertebrate fatty acid synthesis [79]. Both *FAS*, *ACCα*, and *G6PD* are highly expressed in the liver. Hoi et al. [80] have demonstrated that CNE inhibition of lipid accumulation was accompanied by downregulation of *FAS* and *ACCα* on gene levels, suggesting that *FAS* and *ACCα* have a modulating effect on adipogenesis signaling. *PPAR-γ* is a key transcription factor of fat synthesis genes, which promotes lipid storage and regulates insulin. *PPAR-γ* induces fibroblasts or preadipocytes to differentiate into adipocytes [81]. This study showed that CNE supplementation in the diet of juvenile *H*. *otakii* can improve lipid metabolism by upregulating genes for fat catabolism via *β*-oxidation (*HSL*, *CPT1*, and *PPAR-α*) and downregulating genes for fatty acid synthesis (*FAS*, *ACCα*, *G6PD*, and *PPAR-γ*).

## 5. Conclusion

In conclusion, our results proved that supplementation of CNE in the diet of juvenile *H*. *otakii* can enhance their growth, digestion, immune, and lipid metabolism status. Based on curve equation analysis, using CNE at the level of 590.90 mg/kg was recommended as a feed additive in the diet of the juvenile *H*. *otakii*.

## Figures and Tables

**Table 1:** Formulation and proximate composition of the experimental diets (% dry matter).

Ingredients	CNE supplementation level (%)					
	CNE0	CNE200	CNE400	CNE600	CNE800	CNE1000
Fish meal^a^	15.48	15.48	15.48	15.48	15.48	15.48
Chicken gut meal^b^	24.52	24.52	24.52	24.52	24.52	24.52
Soybean meal^c^	30	30	30	30	30	30
Casein^d^	14.8	14.8	14.8	14.8	14.8	14.8
Fish oil^e^	5	5	5	5	5	5
Flours^f^	3	3	3	3	3	3
Corn starch^g^	4	3.98	3.96	3.94	3.92	3.9
Cr_2_O_3_^h^	0.2	0.2	0.2	0.2	0.2	0.2
Vitamin premix^i^	1	1	1	1	1	1
Mineral premix^j^	1	1	1	1	1	1
Sodium alginate^k^	1	1	1	1	1	1
CNE^l^	0	0.02	0.04	0.06	0.08	0.1
Total	100	100	100	100	100	100
Proximate composition (%)						
Moisture	9.92	9.99	10.11	10.12	9.86	10.04
Protein	50.55	50.58	50.61	50.64	50.66	50.60
Lipid	10.41	10.44	10.48	10.55	10.46	10.51
Ash	8.06	8.43	8.21	7.99	8.08	8.11

^a^Fish meal (58% crude protein, 7.2% crude lipid). Shandong Provincial Improved Meiweiyuan Biotechnology Co., Qingdao, China. ^b^Chicken gut meal (65.47% crude protein, 14.80% crude lipid). Liaoning Provincial Improved Yufeng Feed Co., Anshan, China. ^c^Soybean meal (42.5% crude protein, 2.1% crude lipid). Shandong Provincial Improved Meiweiyuan Biotechnology Co., Qingdao, China. ^d^Casein (86.2% crude protein, 1.5% crude lipid). Shandong Provincial Improved Meiweiyuan Biotechnology Co., Qingdao, China. ^e^Fish oil. Shandong Provincial Improved Meiweiyuan Biotechnology Co., Qingdao, China. ^f^Flours (6.2% crude protein, 0.9% crude lipid). Shandong Provincial Improved Meiweiyuan Biotechnology Co., Qingdao, China. ^g^Corn starch (0.3% crude protein, 0.1% crude lipid). Shandong Provincial Improved Meiweiyuan Biotechnology Co., Qingdao, China. ^h^Cr_2_O_3_. Improved McLin Biotech Co., Shanghai, China. ^i^Vitamin premix, 1 kg: vitamin A, 7000 IU; vitamin E, 50 mg; vitamin D_3_, 2000 IU; vitamin K_3_, 10 mg; vitamin B_1_, 20 mg; vitamin B_2_, 20 mg; vitamin B_6_, 30 mg; vitamin B_12_, 0.1 mg; nicotinic acid, 80 mg; vitamin C, 100 mg; Ca pantothenate, 50 mg; folic acid, 6 mg; inositol, 80 mg. ^j^Mineral premix, 1 kg: MgSO_4_·7H_2_O, 5782 mg; FeSO_4_·7H_2_O, 1000 mg; NaCl, 3000 mg; ZnSO_4_·7H_2_O, 150 mg; MnSO_4_·4H_2_O, 50.3 mg; CuSO_4_·5H_2_O, 15 mg; CoCl_2_·6H_2_O, 1.2 mg; KI, 1.5 mg. ^k^Sodium alginate. Shandong Provincial Improved Meiweiyuan Biotechnology Co., Qingdao, China. ^l^Cinnamaldehyde (CNE). Improved McLin Biotech Co., Shanghai, China.

**Table 2:** Primer sequence.

Gene	Primer sequence (5′-3′)
*FAS-F*	CGCTGATTTGGGAAAGGAT
*FAS-R*	GTGAGAGCACGAGGGGTTG
*PPAR-α-F*	ATGACCCTGAGCCATCTAC
*PPAR-α-R*	ACGACAACTTCCTCTTCCC
*ACCα-F*	CCATCTGGTCAGTGTTGCT
*ACCα-R*	TGTTGGTGTGGGCTTATTT
*HSL-F*	CGTTCCTGTCTTTCTGTGA
*HSL-R*	TTGTTTGTTCTTTTTGGGT
*CPT1-F*	ATGCTATGGCTCTTGCTCA
*CPT1-R*	CCCAGACTTTGGTGGTGTT
*PPAR-γ-F*	TCCCTTATTACCCAACAAA
*PPAR-γ-R*	CAACCTCCCTCAGAAACCC
*G6PD-F*	TCTCTCCCCCCCAACAAAG
*G6PD-R*	TACCGCAGCACAGACCACA
*β-Actin-F*	TTACCGTCTTCGGCGTCAG
*β-Actin-R*	AGGGGCATCTTTGCTTGGG

*FAS*: fatty acid synthase; *PPAR-α*: peroxisome proliferator-activated receptor alpha; *ACCα*: acetyl-CoA carboxylase alpha; *HSL*: hormone-sensitive lipase; *CPT1*: carnitine O-palmitoyltransferase 1; *PPAR-γ*: peroxisome proliferator-activated receptor gamma; *G6PD*: glucose-6-phosphate1-dehydrogenase.

**Table 3:** Growth performance and feed utilization of juvenile *H*. *otakii* fed the experimental diets for 8 weeks.

Parameters	CNE supplementation level (%)					
	CNE0	CNE200	CNE400	CNE600	CNE800	CNE1000
IBW (g)^1^	6.10 ± 0.14^a^	6.26 ± 0.10^a^	6.25 ± 0.12^a^	6.14 ± 0.11^a^	6.29 ± 0.13^a^	6.28 ± 0.15^a^
PWG (%)^2^	107.84 ± 3.02^a^	119.56 ± 2.17^b^	129.37 ± 2.39^cd^	130.85 ± 1.87^d^	119.01 ± 2.05^b^	122.43 ± 2.93^bc^
SGR (%/d)^3^	1.31 ± 0.03^a^	1.40 ± 0.02^b^	1.48 ± 0.02^cd^	1.49 ± 0.01^d^	1.40 ± 0.03^b^	1.43 ± 0.04^bc^
FCR^4^	1.49 ± 0.03^d^	1.35 ± 0.03^c^	1.16 ± 0.02^ab^	1.08 ± 0.02^a^	1.23 ± 0.03^b^	1.24 ± 0.02^b^
VSI (%)^5^	12.75 ± 0.36	12.68 ± 0.44	12.52 ± 0.33	12.59 ± 0.49	12.39 ± 0.26	12.37 ± 0.25
HSI (%)^6^	3.32 ± 0.07^c^	3.17 ± 0.04^bc^	2.93 ± 0.08^a^	2.99 ± 0.05^ab^	3.11 ± 0.04^ab^	3.07 ± 0.06^ab^
ISI (%)^7^	3.77 ± 0.11	3.91 ± 0.10	3.89 ± 0.11	3.92 ± 0.13	3.82 ± 0.16	3.87 ± 0.21
SR (%)^8^	82.22 ± 2.22^a^	93.33 ± 3.85^b^	95.55 ± 2.22^b^	95.50 ± 2.22^b^	93.33 ± 3.85^b^	91.11 ± 3.85^b^
CF (g/cm^3^)^9^	1.96 ± 0.06	1.87 ± 0.05	1.92 ± 0.03	2.01 ± 0.05	2.04 ± 0.04	1.99 ± 0.03
FR (%/ d)^10^	1.26 ± 0.01^a^	1.44 ± 0.02^b^	1.52 ± 0.04^bc^	1.59 ± 0.04^c^	1.42 ± 0.03^b^	1.41 ± 0.03^b^

^1^IBW: initial body weight; ^2^PWG: percent weight gain; ^3^SGR: specific growth rate; ^4^FCR: feed conversion ratio; ^5^VSI: viscerosomatic index; ^6^HSI: hepatosomatic index; ^7^ISI: intestosomatic index; ^8^SR: survival; ^9^CF: condition factor; ^10^FR: feeding rate. Values are mean ± SE of three replicates; means with different subscripts are significantly different (*P* < 0.05).

**Table 4:** Muscle composition (g/kg in dry matter) of juvenile *H*. *otakii* fed the experimental diets for 8 weeks.

Parameters	CNE supplementation level (%)					
	CNE0	CNE200	CNE400	CNE600	CNE800	CNE1000
Crude protein (%)	14.15 ± 0.36^a^	14.61 ± 0.33^ab^	15.24 ± 0.24^b^	15.21 ± 0.26^b^	14.97 ± 0.29^ab^	14.76 ± 0.19^ab^
Crude lipid (%)	3.41 ± 0.15	3.51 ± 0.21	3.60 ± 0.19	3.42 ± 0.23	3.57 ± 0.11	3.65 ± 0.20
Ash (%)	2.82 ± 0.18	3.04 ± 0.28	3.04 ± 0.19	2.71 ± 0.31	2.73 ± 0.27	3.01 ± 0.25
Moisture (%)	77.94 ± 0.26	77.97 ± 0.37	77.26 ± 0.30	77.68 ± 0.34	77.21 ± 0.42	77.26 ± 0.27

Values are mean ± SE of three replicates; means with different subscripts are significantly different (*P* < 0.05).

**Table 5:** Digest parameters of juvenile *H*. *otakii* fed the experimental diets for 8 weeks.

Parameters	CNE supplementation level (%)					
	CNE0	CNE200	CNE400	CNE600	CNE800	CNE1000
LPS (U/g prot)^1^	2.09 ± 0.19^a^	2.81 ± 0.11^b^	3.95 ± 0.15^c^	4.32 ± 0.24^c^	4.01 ± 0.36^c^	3.84 ± 0.15^c^
AMS (U/mg prot)^2^	0.26 ± 0.02	0.24 ± 0.02	0.23 ± 0.02	0.25 ± 0.04	0.26 ± 0.03	0.24 ± 0.02
PEP (U/mg prot)^3^	2.83 ± 0.09^a^	3.54 ± 0.08^b^	4.57 ± 0.30^c^	5.35 ± 0.18^d^	4.64 ± 0.20^c^	4.86 ± 0.14^cd^

^1^LPS: lipase; ^2^AMS: amylase; ^3^PEP: pepsin. Values are mean ± SE of three replicates; means with different subscripts are significantly different (*P* < 0.05).

**Table 6:** Apparent digestibility coefficient (ADC) of juvenile *H*. *otakii* fed the experimental diets for 8 weeks.

Parameters	CNE supplementation level (%)					
	CNE0	CNE200	CNE400	CNE600	CNE800	CNE1000
ADC of protein (%)	76.02 ± 0.89^a^	80.48 ± 0.36^b^	82.76 ± 1.42^bc^	85.72 ± 1.60^c^	83.71 ± 1.75^bc^	82.80 ± 1.77^bc^
ADC of lipid (%)	77.16 ± 1.08^a^	85.40 ± 0.52^b^	85.81 ± 0.93^b^	86.11 ± 0.71^b^	87.78 ± 1.75^b^	88.36 ± 1.72^b^
ADC of dry matter (%)	68.51 ± 1.25^a^	76.15 ± 1.64^b^	83.35 ± 2.76^c^	83.37 ± 0.92^bc^	79.02 ± 1.45^b^	82.80 ± 1.77^bc^

Values are mean ± SE of three replicates; means with different subscripts are significantly different (*P* < 0.05).

**Table 7:** Liver biochemical biochemistry of juvenile *H*. *otakii* fed the experimental diets for 8 weeks.

Parameters	CNE supplementation level (%)					
	CNE0	CNE200	CNE400	CNE600	CNE800	CNE1000
SOD (U/mg prot)^1^	218.85 ± 9.57^a^	248.00 ± 6.28^ab^	306.60 ± 10.78^bc^	291.76 ± 13.31^b^	272.69 ± 17.69^b^	292.75 ± 22.78^b^
CAT (U/mg prot)^2^	16.44 ± 0.50^a^	19.33 ± 0.56^b^	22.49 ± 1.19^c^	20.23 ± 0.73^bc^	20.98 ± 1.32^bc^	21.31 ± 0.88^bc^
MDA (nmol/g prot)^3^	2.04 ± 0.20	2.05 ± 0.19	1.76 ± 0.29	1.93 ± 0.16	1.97 ± 0.20	2.16 ± 0.14
AKP (U/g prot)^4^	9.41 ± 0.59^a^	11.29 ± 0.87^ab^	13.89 ± 0.58^bc^	14.27 ± 0.79^c^	12.81 ± 0.87^bc^	13.25 ± 1.21^bc^
ACP (U/g prot)^5^	167.58 ± 7.30^a^	200.28 ± 5.37^bc^	228.23 ± 3.16^d^	201.12 ± 5.45^bc^	197.13 ± 11.65^b^	218.87 ± 6.97^cd^
AST (U/g prot)^6^	18.81 ± 0.31	19.07 ± 0.19	18.85 ± 0.37	18.50 ± 0.22	18.87 ± 0.26	18.75 ± 0.29
ALT (U/g prot)^7^	6.17 ± 0.10	5.98 ± 0.07	6.15 ± 0.16	6.19 ± 0.11	6.13 ± 0.12	6.28 ± 0.17

^1^SOD: super oxide dismutase; ^2^CAT: catalase; ^3^MDA: malondialdehyde; ^4^AKP: alkaline phosphatase; ^5^ACP: acid phosphatase; ^6^AST: aspartate aminotransferase; ^7^ALT: alanine aminotransferase. Values are mean ± SE of three replicates; means with different subscripts are significantly different (*P* < 0.05).

**Table 8:** Serum biochemistry of juvenile *H. otakii* fed the experimental diets for 8 weeks.

Parameters	CNE supplementation level (%)					
	CNE0	CNE200	CNE400	CNE600	CNE800	CNE1000
TP (g/L)^1^	41.26 ± 0.78^a^	45.07 ± 1.33^b^	45.29 ± 1.10^b^	44.82 ± 0.89^b^	45.93 ± 1.06^b^	45.27 ± 1.27^b^
ALB (g/L)^2^	11.74 ± 0.18^a^	12.95 ± 0.15^b^	13.09 ± 0.21^b^	13.01 ± 0.24^b^	11.92 ± 0.20^a^	11.30 ± 0.22^a^
IgG (g/L)^3^	24.34 ± 0.73^a^	26.77 ± 0.39^bc^	28.29 ± 0.43^c^	25.94 ± 0.84^ab^	24.83 ± 0.62^ab^	24.65 ± 1.05^ab^
TCHO (mmol/L)^4^	8.51 ± 0.09^d^	8.01 ± 0.08^bc^	7.69 ± 0.10^ab^	7.64 ± 0.14^a^	7.87 ± 0.09^abc^	8.18 ± 0.08^c^
TG (mmol/L)^5^	2.75 ± 0.05^d^	2.35 ± 0.11^c^	1.93 ± 0.07^a^	2.27 ± 0.11^c^	2.23 ± 0.07^bc^	2.00 ± 0.05^ab^
AST (U/L)^6^	84.29 ± 1.01	83.15 ± 0.89	84.72 ± 1.11	83.83 ± 1.58	84.87 ± 1.46	84.31 ± 0.83
ALT (U/L)^7^	34.25 ± 0.55	34.09 ± 0.77	34.38 ± 0.67	34.28 ± 0.98	34.13 ± 0.78	35.99 ± 0.69

^1^TP: total protein; ^2^ALB: albumin; ^3^IgG: immunoglobulin G; ^4^TCHO: total cholesterol; ^5^TG: triglycerides; ^6^AST: aspartate aminotransferase; ^7^ALT: alanine aminotransferase. Values are the mean ± SE of three replicates; means with different subscripts are significantly different (*P* < 0.05).

## Data Availability

The data that support the findings of this study are available from the corresponding authors upon reasonable request.

## Supplementary Materials

Supplementary Materials Original sequence of lipid metabolism genes for primer design and RT-qPCR expression analysis, which include fatty acid synthase (*FAS*), peroxisome proliferator-activated receptor alpha (*PPAR-α*), acetyl-CoA carboxylase alpha (*ACCα*), hormone-sensitive lipase (*HSL*), carnitine O-palmitoyltransferase 1 (*CPT1*), peroxisome proliferator-activated receptor gamma (*PPAR-γ*), glucose-6-phosphate1-dehydrogenase (*G6PD*) and *β-actin*.
