# Supplementary material for: Dietary Cinnamaldehyde Enhances Growth Performance, Digestion, Immunity, and Lipid Metabolism in Juvenile Fat Greenling (Hexagrammos otakii)
Source: Aquac Nutr. 2022 Nov 2;2022:2132754. doi: 10.1155/2022/2132754 (PMC9973157; doi:10.1155/2022/2132754)
Supplement: Supplementary Materials — Original sequence of lipid metabolism genes for primer design and RT-qPCR expression analysis, which include fatty acid synthase (FAS), peroxisome proliferator-activated receptor alpha (PPAR-α), acetyl-CoA carboxylase alpha (ACCα), hormone-sensitive lipase (HSL), carnitine O-palmitoyltransferase 1 (CPT1), peroxisome proliferator-activated receptor gamma (PPAR-γ), glucose-6-phosphate1-dehydrogenase (G6PD) and β-actin. [file 2132754.f1.docx]

Gene sequence of juvenile *H. otakii*

1. Fatty acid synthase (*FAS*)

TGATTAGCGAGAGACCTTTATTAGTCATAACAATACACACCGGAAATATTACAACACAAGAGATGAGCATACCGTCCCTTCCTTCTCCTTTTCACTGAAGTCAGCCAGCACATTAGCCATTAGACTCTTCATAGACCAGATCAGAGGTCTCAGAGATCAGGTGGGCCCTGCTGCTGCTGCTGCTGCTGGAGTGAGGGTCATTGGAGTCAGAGACGGCTCTTCCAGCAAAGATCCCAGGAAAGTTCACATCAGTTATGTCCACATACGACTTAGTAGAGTCATTCACAACCTAAACGCTGATTTGGGAAAGGATGCGACATCTACCGTCACACTTACGTACCAAAGCTATTCAGTGCATCGCTACGGGTCGGACTAAACACTGCGATAAGTCACCAGTCCTCGACTTCACAACCCCTCGTGCTCTCACAGGAGTCGGTCATTTGTTTTGGGGGCAGAGCGAACACTGAAGTTCGTCCGCTGGGAGGAATGGGCCTGGACTCTGTCGCCTATCAGACTGCTGAGGTTTGCCCATCAATGAGTAAGCCCTGTAACAAGTCATCTGTCTGGCAAATGCAACAGCTCTTCCTTTCTAGAAGTGTTAGTTGAATTACTGCAGGATAATAATAATAGTAACCATTGGTTAAAAAAAAATTAATAATTACATTATATTAGTAACAGGGTATTTTTTGCGGTATTGATCATTCTTTACACCCTAAATTAAAAAAAGTTTCTCCATGTCCTGTAAACAGCATCTATCTTATAGATCAGTGTGGTCTATAGAGGTCATTCAGTAACACTCTTAATTATACGACATTGTGGAAACACTTTTTATGACATACTGCGTTTACAGCTGCACCACATGAATCACAATGACTGGTTGTTGTTTTTAGAAGCTTTCCATAATTTGGGTGAGTTTTGGTTCGGATCAGGAGAGTAACAGCTCCCGCTTCGCGTCGAGACAAAAGATGATGAAAACAAAACAAACAAACAAAAAGTAAAACAACACGTGTGATTGCGTCCCCTGACACACAGGTAAACAAACTTCTTTTGGACAAAATAAACAAATAATAAATACATAATTAAAAATAGAACAAACTCCAAATGGTCATATTACCAATGCTATCAGGTATGTAATGGTGCTAGGTCAGGAGTAATTTAACAGGTGTGTGTGTGTGTGTGTGTGTGCAGATACAGAGAGAGAGAAGCCACACAGCTACACTAGCAGAGCTCGGTTGTGGGTCATGAGTGGACCGTCATGAAATAACCCCGTCTAACGGCCGAGGAGCACAGACGCGACGCGCAGCTGACTGCGTGTCCGTTGCTGAGCAGGAGTCGACCTTCTCATGCTATGCTAAGCTAAGCTACATTATTTCCTAGTTGTTTAGGCGTTAGGAGTACAGGTGGAACATGCTGTGTACGATATTCTATTAGTTTAGACATGTTAATTGATAAAAGCTCATCATTGAATTGGTTTAAAAAGCAATTTACTGACGCAGCAACTCATGCCAAAAGGGAGGGCTCTGTCACGAACACCTTGGTGATGGTCTCCGCCGAGGATGAATGATTGAGAAGGTTTCAGAACCTGCTGAGCAGTATTTTGGCGTCTGATCAGCCTTCGAACAGCTAATCACACACTTGTGTGTCTAACCCAGTGCTGTTCTTGGTACAGGGGGGACCGTACAGGGGTTCTACTGTATTGTGTTGAGCCCTCTGCCCACTTGTGGGGCTCTTATGGGTTTGAAGGGTTCTAGAGTGGGCTAGCCGTGGCCTGCTCTCTGCGGGGGTCTCCAGAGTAACAGGTTGGTATGTGTACGATACCTAATGTAGCCTATAGCCTCCCTAATCAATACTGCCCATGTAAACAGTGAAACATGTTGCGTTTTATGAATGAATAATTATGAATGACTTATGGGGCTGGAGCTGACTCTGAAATCGGGATCCCTGCTACGATCCAAAACCAGGCTGAATGAAACACGGTAATGTAATGAGACAGAACTTAGGACTTCACATTTCATTTAGCAAACAGAAGACATCAGCTCGGCGCTCTGAGAGGTCAGGGAGGGAGTCCAATGTTTCAATTGACCAAGGAGATTTATAAAAGGTGGAGTAAGGTGCGTTTGGGGTATCAGGGCAAAATGGCACCGAGCGCAAACATGCTGTGTATTATTCTCACTGTATAAAACAAAAGTTGGGAATGTTGTGATATTAGTGGGAAGGTGGGGGGGGGTTGGGTGGACATGTCTAGCCCTCCCTTGCTGTGACCCGAGGCTCAGCCAGTGAGCTGTGGATGATGCTGCTGATGGACTCCACCCCCTCCCCTTCCAGGAAGGTGCGGTGGTCTCCCTCGATGACGTGGACCGACACCTTGCCATCGCACACCTCGCTGAGCTTGTAGTCGGCTCCCAGGTTCTGCTCGTATTCACTGCTGGTTTTGGCACGCAGTAGCGTCACGTTGCCGCGGAGTTTTGACGCAGGCACGTAGCCGTCAGCCGCCTTCAGCTTGTAGTAGAAGGTGGTGGCCGCGAAGTGCAGCGAGTCTCGGCTGATGTTCTTGTGGCTGGAGGTGATCAGGTCCACCGCGATGTTGACACGGGCCTCCAGGTCCGGCAGCGGGAGAAGAGTCTCCAGAAGCTTGTTGTACGCGATGCCAGTGAACTGCTGGATGAAGGCACACAGGGCTTCCGTTTCAGCCTCAGACTCTTTGCCCGGTGTCAGCTTGGCCCTGTAGCTCTGCGTGTACGCTGCCACATAAGAGTGTGATCCATCAAACAAAAAGAGGTACTCCACAGGCAGATTCTGGGTCTGAAGCTGGGAGCACATTTCAAACGCCACACAGGCACCAAAGGAGTAGCCGGCGATCCGATAGGGCCCGTCTGGCTGGACCTGTCTGATACAGTCCACATAGTAGGCTGCCAGGGACTGGATGCTGTCCAGAGGAGCAGCTTGGGTGCACTGCAGGCCGTAGCAAGGCATGCTCAGCTTGAAAGCAAGCGTCTTAAAGGCAGAGATGGAGCCCTCGATGGGATGGACCAGGAACAGCGGCCTCTCCTGGTTCTGGACCTTGTTGAGTGGCGTCACCGTGGGGCCGCTGGGGTTGACCAACAGCTGGGTCAGATCAGGCTCCAGCAAAGAGCGAACGGCGTCCCTCTTAGCCGCAGCGGTCTGAGATTCATTTGATCCGGCGGGCTTGCTGTTGGCCAGCTCTCGTAGCTTGTTGATGGTGACCTGCCGGATCTCCCTCATGGCCATGACGATGTCGTAGTCCCGCTCGAGAGTCTGGCGAACCTCAACGCCCATCAAGGAGTCTAGGCCCAGGTCAGCCAAGGAGGCATCAGCGTTCAGGCTGTTCACATCCCGCACGCCGAGGATGTGAGCGACGGCGTCCACCAAGTCTCTCCGGCTTCCTGCCTCGCTCTTCACCGCCGCCCTCTCGGCCAACACGAAGCTGGACATCACCGGCCGCCGCTGGCACAGGAAGAGGTCGAGCACCTCCAGGCAGGAAGCGATGCGCTGCGGCAGGGTGCCGCCGATCACCGCGTCGTTGCCGCCCATGGTCTCCAGCACCACGCCCACGTCGCCGATGGCGCCCCACTGCACCGCCAGTCCGGGTAAGCCGTCGTGGCGGCGCCGCTCGCACACGCGCTCCATGGCCGAGTTGGCGTAACCGTAGTTACTTTGGCCGGCGTTGCCGCGGCCGCAGCTGACCGAGGAGAAGGCCACGAAATGGCTGAGGTCGGGACACGAGTCTCTGGTCACTCGGTCCAGGTTCACGGTGCCGTCATATTTCGGTTTGTTTACGTCGAGGAAGAGCTGAGGAGTCAGATTCTCCAGCATGCCGTCTTTCAACACCATGGCGAGATGGAAGACGCCGCCCACCGGGCCCAGTGAGCCGGCCTCGGCGAGGAGTCGCTCCGCTCCCTCCAGCGTGCTGACGTCGTTGGTGGACACCAGCACCTCCACGTCTCGACTCTGCCATTCACGCACTCGCTTCGCCTGGTAGCCGTTCCTGATGCCGGATCTGGAGGTCAGCACCAGTTTGCGGGCTCCTCTCTCTGTCAGCCACTGAGCCAGCTCCAGACCGAAGCCCCCCAGTCCACCGGTGATGATGTAGGCGTGGGAGGGGGGGCAGAAGGTGCGGCAGATGGCTGGAAAAGACAAAGGGGGAGCGATTTGGACTGCTGAGCCCTTCTCCTCATGACAGACCTGCAGGAGGACCTTGCCAATGTGTTTGCCCTGAGCCATGTATCGGAAAGCTTCCTCCACTTGGTCCCTTTCAAACACTGTGGTCTTCAGAGGCTGGACTACACCTCCCAGAATGCCTTCCTTCAGCAGCTGGGACACTTCCTCCCACTCCCGGTTGCCCTCTTCAAAGAGCGCATCCAGCAGGATGCCGTGGAACGCCACGTTCTTCAGGAACAGAGCCATGCCCAGTGGGGAGTTGTTGGACAGGTCGAATTTGCCGATCTCCAGGAATCGTCCATGTCTGGCCAGGCAGCGAATACTAGCCTGTAGCTTCTCCTCAGCCAGAGAGTTCAGCACCACATCCACACCTTTGCCCTGTGTGTGGAGCAGGATGTGCTGTTCAAAAGAGGAGTCTCTGGAGTTCGCGAAGGACTCTGCTGAGAGCTGAGGGAACCTCTCCTGCAGGTAGGCCCTCTTCTCCACTGAGCCGACGGTAGTGAAGACTTTGCAGTTCTTGCTGAGTGCTATGGCGATGGCAGCTTGGCCGACCCCCCCTGACCCTGAATGGATGAGAACAGTCTCTCCGGGGCGGAGCCTGCCGCGCACCACCAGAGAGTAGTAGGCCGTGGCGTAGACCACCGGCACCGAGGCTGCCTGCTCCAGTGTCCAGCTGGAGGGAACACCCCAGAGGAAGCGTTTGTCCGCATCCACACTTGTTGCCAGGCCTCTGGCAGGCAGCAGCCCCATCACACGCTGACCCGTGGGGTCGCGACCGGAAAACTCCATACCCAGCAAGCACTGCTGCAGAGCTAGGTCACCTGGAATAGCATCTGGTGGCAGTTTGCCGGTGGCCAGCATTATATCTCGGAAGTTGAGCGAGCTGTAGTAGACGCGACACAGCTGCACGTTGGGGTTGCTGGTCACAAAGTGACGGAGCGGGGAGGCGATCCAGCGCAGAGAGGAGAGATCACCGCGAGTCAACACGTTGACGTAGGCCTGCTCCGTCAATTCTTCGTTTAGATCTCGAGTGATGAGCTGGTGCCTGAAGACGCCCCACTCTCCGTCTCTGAACACGTTCATGACCAGATCCCCGTCCAGCACCGACCGCATGGACTCGTGGGCTGGCTGGAGGCTCGGCGCTGATGAACGCTCGTTTAGATTGGATACAAATGCACAGTGTATACGGTTGCCACCTGGCTCCTGCCGTAGACAATTGACCATTCCCACAATGCCGCAGTGGGACTTAGAGGCAGTCAACCACACTGGACAGTCTGATGATTCTGCCATCTCCGCCTTGAGTGTTTCCACCCACTTGTAGTCGGTGCCGTCCACAGGTAGGAACACGGGCTGTTTGCTTGGAGACCGGCAGCGGCACAGGAAAAGGGCCGAGCCATAAAAGGACTTCCTGACAGCCACCAGGCTGAGGGAGGCCTCAGAGAACACCTTCTCCCACTCGGCCTGTGTGAGCAGTCCTCGCTGGCTGCTGCTCTGAGCATTGCTAGAGAGGAAGGCAACCGTTTCCCCCAGAGTCTCTCCCTTCAGCAGCGTGTGGAAGAGAACAAAGCCCCCTTGTTTGGCTCCAGAAGCCAGGTTGGCCACTAGCAGTCCAGGGTCCGTACTTAGAGGGCCCCAGGCGTGGTTACACACCACGAGGTCTGCCCCGCCTACAGCGCCTCCAGGAGCCGGGCCCGTCACAGGGTCCCACTGAGCCGATGAGACCGCCAGCTCCTCCAGTGTGGCCTGGTGGGGGGTCAGAAGGTCCAGGTTGGTCGCTGTGGCGGTGTAGTCCACACGCAGCATGGGCTGGATGTTGAGGAGCGCTACCATGCGAGAGAAGAGCTGGCCATCGTTGGAGAGCGCCTCCAGGACTTTGATCTTGCCGGGTGTGCTGTTTTCTACGGTGGTATCCAGGCAGTGTCTGAGTGCTGGGCTGTCCAGCAGGCCCTGCAGCAGGCCGTCCTGCAGCAGACACGCCCGCTCCTTCTCCACAGTCTGTTCCAGCTCGGATCGAAGGTTCCCGTTCAGCTCCAGGCCGCAGAGCAGAGCCAGCAGCCGCACCAGGCCGGGCTCCGAGGGCTCGGGGCTGGGAAGTGGGCCGTCTGACCCCCCCTGTAGTCCGGGGATGGACAGTTTGACACCGTGGAGGGCCAACTTTTTCTGCAGTCTCTGGGTCAAACCTTTACAGAGCCTCAGCTGCTCTGCAAGTTTCCCATTGGCTGTCAGGCACTCTGTATCCACATACGGAACAAACACAAACTCCTCCAGGGTGGGGGGGCTCTGCTGCTGCTGTCGACGGGGGGCCACAGTGGCGTGAAGACCGCAAATCTGGACTCCACCGGCAACAATGTTGTCGAGGCAGCGGTTTACGTGGACATCCATCGCTTGCTCTCCATCAGTGTGCTGGCAGACCTTCTCCAGGTGGACAGCTGGATCAATACACACTGAGCGGATCCTGGTCGGCAGACGCAGACTACGACCCGACAGCCCGACCACGATCATCTGCAGCATGGTGTCCAGAAAGGTCACCCAGTTTCCGGTCCACTGCAGTTTGCCCCTGTCTCCTGCATTGTTGGACTCCAAGATGCCTTGGAAGGTCTTTCCGTAGTCATAGCCGCGCAGTCGCAGCTCTTTGTAGACATCATGTGCCGTAAGCTTCATTTTGGGGTCGTCGTCGATGGCAGCTTGCTGACTTATATGACTATGGAATGAATCGAGGGCTGCGTCCTCCAGAATGCTCACCTTACCACTGACTGCCAGGTTTCCATTCTCTGAAACCTCAAACCGGTTGGTGGCAGGCATGAGATGCACCTTGAGCTGGACCGAGCCGCTCTTTGGCAGGATGGTGGCCCTGTGGATGGTGATGTCCTCAAAGGTGACGGGGGTGCTGTCCATGACGACCCCCAGACTTCTCACTAAGGTACGCCAGGCCAGCACCAGGTAACCCGTCGCCGGGTACAGGACACGCCCATCAATGCGGTGTCCAATCATGTAGTAGTCTGGAGACTCTGGGTTTATGTCGATATTATAGACAGAGGCCGAATTGGATCCTCCTGAGCCACAGGAAAAATCCTCCACCTTGGGGACATCCCAGGTCTGGGCGTGGTCCCACTGCACCAAGGGGGATATCAGAGGGGTACCGACCGGCACGGGGTACTTCACCACCGGGCAGAGGCCGTTACCGTCCACACTGATGCCGTTCATGTAGACTTTGCCGATGTTTGAGAGGAAGAACTCGAGGTTGTTGGCGTGACCTCTCTTCATCAAGGGCAGGATGGAACACGTCTGCTTCAAACTACGCTTCAGGATGGCCTGCAGCAGAGCGTGAGGTGCGATCTCCACCACCACGGCGTTCTCAGGCACCAGACTGAGGCCCTCGTGGAACAGCACGGGGCTCACCAGGTTGTTGACGTGGTAGTCGGCCGAGCTGAACTGAGCCAGAGGAGAGTCCCACTGAGACTGAGGGATGCTGGTGCTCACCCAGCGGGGGGAACGCTGACGTGGCTCCTTGATCACCTTCTTCAGGGCAGCCAGCAGGGTCGGAGCGATGGAGGCCATGTAGTAAGAGTGGAAAGCCACACCAGCGCTGCGCACCTCCTTTGCAAACACTCCCTGCTCTTTCAGCTTGGACACGAATGTACTGATTGCTTCCTGAGGACCGGAGATGGTGACGGTGTCCTCAGAGTTGTGGCAGGCTGGGACCACTCCCTCAGGACACTGAGCCATGCACTCCACCCAGGTCAACCCGACAGCAGCCATGCCTCCTGGAGGAAGGTTGGCCTCCTTGATGCAGCGGCCTCTCCAGTAGGCAGCCAGGATGGCCTCAGTGTGGCTGAGGGAGCCGTCAGCGTAGCCACAGGCCAGCTCTCCCACCGAGTGGCCCACGATGCCGTCGGGCTGCAGACCCAGCTTACTGAGCAGGTCGATCTCAGCTATCTGTATGGCGGCGAGGCCGACGAAGGCGTGAACAGTGTCTTCAAAAGTGGCTTCGTCTGCGTCCATGAGGAGGCGAGACACGACCAGGCCGGTGTCCTTCAGGGCTGCGTCCGACCGCAGGATGGACTCTCTGAAGTCTGGCAGCTGCATGAGACTGCGGCCCATACCGTCCCACTGTGTTCCCATACCTGAACAGACGTACCAGAGCGGCCTGGCGGTGGCCTGCACCTGCTGCACCTCCTTGACGTCGCTCTGGGAGCCGATCACAGCGTAGCCTCGGTAGGGCATGCTTGCAGCGGGAGCTCCTGAGAGCTCATTCAGCAAAGACAGGAAGCTGTCGTCGGCCGCGTGTTCCTTCCCCTTCTGGAGCATGGCCTCCACGGCGGCCTCCGTGCGCCCACAGGCCTGGAGCAGCCTGGGAACCGTCCTGGGCGGCGCGGTCGCGTCGGTTGGTTTCTCAGCCGGGCGAAGGATCACGTGAACGTTTGAACCTCCAAATCCAAAGGAGTTGATGCCTACGATGCCGCCTCGGACGGGAATAGGCCGGTCAACGACTTGAACCCGCCCGTCGGAGAGCGCTGGAATGTCAGGGTTCGGAGAGCTGAAGTGCAGGTTGGGAGCCCAGAGTCCTCGCTCCAAAGAAAGCACCACCTTAGCCAGAGCAGCCAGACCAGAGGCCGGTTCAGGATGGCCCATGTTGGACTTGGTGGAGCCCATAAGCAGAGGCTCTCGCTCCGATTCGCAGAAGACGCTGACAATGCCGTTCACTTCCTGCGGGTCGCCGACCTTTGTTCCCGTGCCGTGGGCTTCCACGTACTCCACCTGCTCGGGAGATATATTCGCCTCCTGGTACAGAGATCGAACCAGCCTCTGCTGCATCTCACCCGAAGGGAACGTTACACCCTGCTCTTTGTATCCATCTGTGTTGTTGCCAGCGTTGACCACTGTGGCATAGACCCTTTTAGCCGCCGACCGCTTGGTCAGCAGCACCGCCACAGCAGCCTCAGAGCGGCAGTATCCATTTCCTGATGCGTCAAAGGACTTGCAGGTCCCCTCAGGACTGAGCATGCCGAGCTTCATGAACTGCACCGAGGTGTTGGGCTTGAGCAGCAGGTTGACTCCTCCCACCAGAGCGGCATCACAGTGGCCCTGGCGAATGGCGTGGAAGGCGTTCTCTAAAGCCAGCAAGCTGGAGGAGCAGGCTGTGTCGATGGCCGTGCTGGGGCCGCTGAAGTCGAAGAAGTAGGAGAGTCTGTTGGCCAGCATGGCGCGCTGGCAGCCCGTCATGCTGTAGCCCAGCAGCTCCTCTGGATCTCTGCTGAGCGCCTCGCCGGCCTCCGAGCCGCTCACCCCGATGTACACGCCCGTCTTACTGCCACGCAGCGCGGCCGGGTTCAGTCCTCCATCTACGATGGCTTCGTAGGCGATCTCCAGCATGAGACGCAGCTGGGGGTCCATGGTGTTGGCCTGTTTGGGGTGGACTCCGAAGAAGGCTGCATCGAAGTGGCTGATGTCCTTCAGCTTACCGTTCCTCTTTGGAAGACCGTGCAGACCTGGTGTCCACCGCCGGTCGTCCTCCGTCACCATGTCCACACCACCGATGAGGTTTTCCCAGAATTCCTCCAGGTTGTTGGACTCGGGCAGACGGCCCGATATCCCTGCTATGACTATCTCATCCATGCTGAATTACGGTGCTGCCCGGCGGTGCTGCCTGTCTGTCGCTGTCTGTCCCGTCGGTCCCGCTGGTCTGTCGCTTAGCGTGACTCCGCTCCTCTCGGTCTCCTTC

1. Peroxisome proliferator-activated receptor alpha (*PPAR-α*)

CGGCAAAATTTTATTGTACACTACATGATACAAAATATAAAGTTTCATTCTGACAAGAAACAGTTTTTCGATACTACATCGGGGAGGTGAAGTGTAACTCAGCAAATGAGGCAACAGTTTTACTGTCAGCAGACACTTTCACTGTGGCAACCACATTGTTATATTTACAAAATACCACATAAACCAGCTTATTGTGTTTGTGGTCAAAATATTCACTGGCCAATAATGAGTCTTAACGGCAGGATTTGTACAAGAAAAACCCGAAGTAAGTTGTGCTGTCATGTGTTGGTTTTTAGCAGTAGGTTAAGGATTCAAATAAATATATTACACATGAAATTACAATATTTTCAAACAATGGTTGCAGTCAGTAGAAGTAAGGGTAATTTTTGTTCATTGCAGGCTTAAAATGCATTCTTGTTCATGATTCATGATGTGAGAACATTCCCTGATCCAACACTAAATGTCTGTTTTGTTGTGTGGTGAATGTGTAAGGTGTGGTCAGCATATAGGAAATATGAGCTCCAGCATTGAAACATTATGGCACATGGTGAATACTGATGCAGGTGTTTCTTCAACCACTACGTGGCTGATTTCAATAAGGTACTAGTACACAATGAAAGGACATGACAAAGCTGAACCTAATCCAATCGAGTTGAATTAACGCATCACATGCAATTATCCAAAATAATAGGCCTATTTTGGTATTGAGTTGCCTATTGCATAACCAACATTTCAGTTTCCTAAAGGATTAAAATGTTTTAACTTCTCTAACTTGTCTGCTTCTTTTACCACCATTCAACTTATGTTCAAGATTAATATTTAGCATCAGTGCATGCATGACTGCAGCAGCCATGTCCGGGTATCTTCTAAATTTTTGTACACCCATTTTAAATGTTATGCAAACTTGCAAAGTCTAATCCCTATTTAGCTTTTGATCAGTCTGTAAAAGTAAAAGCTGGCATCCAAGACCCAATCCATAGCAATTGGGTGCCCTCAGATCAATGCATTAAAAAAATGATCTCTACACATCTATGGTGTAAGATCAAAAGGTTTGTTCCCCACGGTGTCCCCTTTCTTCAAAGCAGCCGCGCCACCTACAAAAGCTGCCTTTCCATTGCAGATCACAGCAGTGTGCACCCTCAGGAGCTGCACAATGTGCTACTTACAGTACAGTGATGTGCATGATAGTATCAGCAAATATTTATGGCCACCCAGTTCTGAGTGGCATACATAAATATTCAAATACATGTCCTCGTTCATGACCCTGAGCCATCTACTCAGTACATGTCCCTATATATCTCTTGCAGGAGCGGGTGCAGCGACGTGTCCTCTGTTGTCTTGATTTCCTGCACCAACTGAGCGTGCTCGGTGACCAGCTCCCGGAGGTCGGCCAGTTTCTGTAGCAGTCTGGGGAAGAGGAAGTTGTCGTCCGGGTGGTTGGCCAGCAGATGGAGCCGGAGGGCCTGAACGATGCTTTCCTGCAGCTGCTCCACTAGAGGCACATCCACCAGGCCTGGGCGATCTCCGCAGCAGATAATGGCAGCCACAAAGAGGGCCAGGTCACTGTCGTCCAGCTCTAGGGAGTTGAAGCGTGTGGCAAACTGGAATTTGGGCTCCATCATGTCACTAAATGGCCGCCGCCGGCTCTTGAGGAACTCTCGGGTGATGAAGCCTCCACCACGGGCCACCAGGAGGCCGTCTTTGTTCATGCAGGAGGCCAGTAGGGTGAAGAGGGCTTCATAAACGCCATACTTCAAGAGAGTCACCTGGTCATTTAGATCCAGGTCTTGGAAACCCGGCACCGCCTTGGCGAACTCCGTCAGTTCTGTGACCGTCTCCACCGAGGTGCTCTGGCAGCAGTGGAAGAGCCTGGCTTCAGCCTCCCTCTGCTGGAGTTCCCCACACCCTGTGGCCAGAACCACCTCCCCAGCTTGAAGGCCACTCTCGGGCTCCGGACAGTCACTGTTTACCACCTGGGCCGCTAATGTCCTCTCTGCCAGCTGGAACGTCTCCATGTCGTGAATGATGAAAGGCGGCTTGCTGGTCTTTCCGGTGAGTATGAGCCGAGCTTTCGCCTTGTTCATGTTGAAGTTCTTCATATAGGCTTCGTGGATTTGCCCCACCAGAGTCTTATGGTCGGTCAGCATGGGGCTTGCCACCTCTTTCTCCACCATCTTGCATTCTGCCTTTAGCTTTAGCTTCTCCGCCTGAGGCATCCGACCAAACCGGATGGCATTGTGGGACATGCCTACAGAGAGGCACTTGTGGAATCGGCAGTACTGGCACTTGTTGCGGTTCTTCTTTTGGATTTTGCAGTTGCGTTCACACTTATCATACTCCAGCTTCAGCCTGATGGTCCTCCTGAAGAAACCCTTGCAGCCCTCACAGGCGTGCACCCCGTAGTGGAAGCCCGAGGCCTTGTCCGAGCACACCCGGCACTCCAGGTTGAGGGGGCCGACGCTCTCCTCTGGACTTGGGGCTGCCACACACACCCCCGACGATGGACTGGAGGCTGGGGTCAAGGTGTCCAGTGCAATGGAGCTGTCAGACCCTGAGCCAGTGCTCTGGTACGCCGGGAAATCAAATCCCAGAGTATCGTCTCCTATAGACTGGGAGATGTCACGAAGATCCTCCATCAGGTCCCCACACAGTGGACTGTCAAGCAGGGAATCCCCAAGAGGGGATGGGGGGCTAAAGAGATCCCTGGCCATGGTGGAGATCAGGCCAAACCTGGGAGGAACAAGCCCTCACTCACCAGACTCAGAGGATGCCACCCGAAGCATTTCCAGATGCTGCTGCCGGCATGGCCGCCCCTCCGGTCCCGGGACGCTGCGCCGCTCCACTCGGTT

1. Acetyl-CoA carboxylase alpha (*ACCα*)

CCTGTCCCACAGTCCACTCACCCAACACCCCAGCAAGCCGCCGGAAGATTTCTACAGTGTACACGGTGACACACACACAGAGGTGGATGCACTGGACCCTAGCATCATGGACTTTGCCCTTCAAGGTAATCTGTGGGAGGAAATGAAGGACGACAGCTTTAACCTGGATGCTTTGGGTACCTTCAGTAACTCACCCCTCCGACTATCAGACTGTGACTTGGGATCAGCCAACCTGACTTCTGTCTCCACTGGAGCGCACCTGCCGCTGTCAGATGTGCAAGTGACGGGCCTCTACACCTCCTACACCTCCCAGGACCACCTGTCTTCTCAGTACATGGGCGCGCCAGCCAACAGCAAGCCCATCGCCCTGTTATAAAGCAGCTGAACTGTCTCCTGCAGCGTCAACCTGATTCATTTCACTGTCCGTGGATATCTGTGGACGCCCACTGAAGAAACCTGAGGTCAAAGTCTGATTTCTGGTTTCTTTTGTGTTTTAAACGTACAAATCACACAGCTCCTCTAAAGTCAGCTGGGAACTACTTGGAGAATGATGCTTGAACATCATTTCACTTGCGGTCCACAAGAAAATAGCTCCTTAAGAACTAAAACTGCTGATAAATAAAGACGTGGCTGTAATGGTATGTAAGACTGATAATATCTAGCTTTGCCGAGCAAAGACTCAAAGCCTGTATGAGAACAAAGACGACATGGAGATGTACGTGTAAGTTAGCATCTGCCAGTTTTTCTACTGTTCACTCTGCGTTTAAAGGCCCTACCATACTCTAGCTCCCCATCTATTGTCTGTTGTATTGTGCTAGACATTATTGCTGTGATGTTTTATTTATAATTTATGAAAAGGCAGCTGGAGAAGGTGATGACTCTATCAATCTTTTGCTGTATATATTTGAATGTTTCAAATTGCTATCGCCACAGAAAAGCTTTCTTTCGACTGTATTTGCCCACTTCATTCCAAAATGCCGATGTGTGCCAACACTTTGAATAATCCAGACTTTACACATTATTCTTTTACACAATGTGATTAACATGCCTCAAAGTCAGATGTATATTATGTTCATTAATTTGATCATGATGAAAATCAAAAGGGGTGAATAAAAGAAGTTTGTTTTGCCAAAAATATATCAATGACATTTATTAACAGTCTTCAGCAGCATACAAGACACAGGACAGAGTTTATATACATTTAAATTAAATGTCACTGTTTTGATCAGAAGAGAGGCAAATTAAGCCATTGCTCCGGCAACCACTTTGGTGATTATTGTCCAACCTGAGTCTTAAGAATTAATGCATTATATCAGGGAAATACCAACAAAAATGAAAGAAAAGCTCAGAAGTATCACATAAAGTGTCACAGTATCTCCTATCATTCGTTACATTGTTTTAGAACTCAAAACACGACAGCTAGATACACAAACAGACTGAAAGTGACAATGAAAAGATGGACGGCTGAGTTGGACGACTGAGTCCATAATACTATTTATTTATTTTTTAAATGCCTTTTTCTGTTCTTAATTTACATTTCTGCATTACAATACTGGTAAAAATATATTTTTAGTCAACGCAAAAATACTTTTCTTTTTTGTTTGTTTTACTTGAGAGTACTGAGCTACATTCAAAGTTCAACAAACATTCTCTATTTAACTCCTAAATATAGAGTCTCCGACTTCATGAAGCTTGTGCAACTCTGGCCTCAATTGACTGAACAGACCTCGTTACACTGTCACACTATTATGTGCAAATACAACAAAATGAAATGTGCGTTACAATCATAGTTTGTGAACCAGAAAGTTCCTGGACCGCGCAGCTGAAATGACTGACTGCTTACCTCACAAATGCAAAAGACAAACTCTTTGTCTACCTGTCAGTCAAGAGTGGAACGGATCATGATATCCCTGCAAAGACGCCATTCAACAGCAATGTTTGCGACAGACAGCTGTAGTACAGCACTGTAGTGGTTATGTGATCGCTGAGGAATAAGACACTTTGTGCCCCAACTATACCCCGAAAGAAATGCTAGTTTTCCACCTCTTTACCACACATTACAATCAACTAGAGCAATGTTGTTTGCTCTAATTAATAATATAACCTGATAAAGCTCCGCAGCTTAATTGGTGCTGGTGCTGTCCATAGTTGCAAGCAGATGTGAGAGCTTGGCCCTCTGGGACTGAGTGATATTCTGGCTCATGTGGATGATGCAGTCCATGGCTACGTCTGGGTTGGCCTGCACCAAGCTGGAACACAAGAAGAGCATGCTGGAGTTAATCATTGTAAGCGCAGAAAATGCAGAGATAGTGAAACTGTTCTGAGCTGGTCTGCAGTATATTCTGTCCTTTCTGCGTAAGAAAATATGACTTGCATGTATCTCAGTGAGGTCTCAAATATTGGATGGACTCAACAGCTAAAATATTCGGTTTGTTTTCCATAAATGATTTAAGATGAAGCCATCTGGTCAGTGTTGCTGTCGTGTTATGTTCTTCAGGCCATGACCAACATGACCAACATGACCAAACTGATGGAGTGATTGTGACTCTATTAGCTCGTACTTTATCCATTTATGTCCACAATGTAGCTAAAATCATTTTAGGATTCATTAAGATGGGACATGTGTTTTTTTGGCATTGATAAGATTATTGTAATTAAAATAAGCCCACACCAACAGGTCACAAAATGTGTGTGAGTGTGTCTGCATACTTGCGGATGTGTTTCAAAGTGTTTTCCCTTTTCAGGTACTTGATGTTCTCTCGGATGCCTGATCGTACGCCATCCTCCTTGGTTAAATGCTTCTCGAGCCACTCAACTACTGCTTTGTTATTATCCCAGAGGTAGGCCTTGACCGTTCCTTCCGTTTCAACAAACCAGCGTCGAAGCATGGACTGCATGTGTCCATCACTAAGGTCTTTGTTGGCCTGCAGAATCTCACACTTCACCACCTGCTCCAACAAGAGCCGACGCAGACGCCAGTAGAAGAAGGTCCGCACGTTCTTCCAATCCAAAATATCAGTGATGACACCCTTCTCCTGCATCCTCCCTGGGGTGTCATGGAGATCTGCGAACTGCACTGCCACCTGGTGGTAGATGGGCAACAGGAACTCCTCCCTGGCTTTGAGCTTTGACTCCAGCTCTCTGCACTGTTTGTCAGGCAGCTCTGGGGAAGCAAGCTGCTCAACCAGACTAGCATAGACTGAATCTAGTCTTCTCATGGTCTTCAGTAGATCCTTCCTCCTGAACTTGATCTCCACTGTACCCTCAGCCTCCAGCACACCACCTCTGCTCTCCCTGTCTGCATAGAGCTCCATACACAGTGGGTTGATGGTGGGGTCTATCACCACCCATGATCCTCCTCTCAGCTCAGCATGTGGTGGGATGTACACCAGCACCGGCTGATGGAAACTACGCAGGGCGTCCACAATGTAGGCGCCAAACTTCAGTATCTGGTCATACATATCTTTCATTCCACCAGAGAAGCCCCTCCAGTTGGCAAACACCATGAGAGGAAGATGTTCACGGTTGAAGTCACAAATGGCCTGAGACGTTTTAAAGGCTGAATCTGGAAACCACACCTGGCCCGCTTGCTGCAGAACTTTAGATTCTGAATCCAGGTTTGCTGGATCCGCTGGGACAGTGTACTCAACTGTCCGTGTTTCAACGGCGATGACACCAAGGGGAATTCCTCCTAATCGTGCTCTGCCCACTACCACCGTCTGAGCCCAAGACTCCATTATCTCCATGAAAGAGCCGTGGTCGAAGAATCCACTCTGCCAGGAACCTCTCACCGTGGGGTGAGGTCTCCCAGCTAGCATCCAGCGGGGGTTGTAAGGTGCTTTAGTCGGAGTATATTCTATCTCTCTGTCTACTGGATCTGTCGTTGATATGACAGGCACAGGGGAGTGTTTGTTCTTTGGCATGTAGGAGAGCCACTGGAGGATGGTGAAGACGCCCTCAAAATCATCTGGCACGATGGTGTGCGTGACTCCATTATTATGCATGATCTGGACCCCTCCCAGCTGGTTGTTGGATGTATAGACCGGCTTGCCCAATACCTTGTTCAGAGCACCTGCTCCAGTCAGGATAATGTGAGAGTTTTCCACCTGAATCACTCTCTGTCCCAAACGGACCAGATAGGCTCCGATTCCGATAGCGCGGCACGTCACCATACTAATTGTAATAATCTCCTCGTACGCCTGAGAGGATTCTCCAGCAATGGTGCCAGAACCTCGCAGGTTCTCAACCCCAAGACCTTCGTCCTTCCCGATGATATCAGTGATGATGTACCTGGACTCTCCACCTTCCTCTACATGGTGACAGTGAACAGCGTTGGTGGAGCTGATACGAGTGTAGTCCTGCGGTGTCAGATAGAGGTACTTGATGCCCTTGTAGAGGTCAGTGGGGTCAACCCAGGCCACCTGGAACATGTGTTTGATTTCTTCAGCGAGGCCAATGCGTGCTCCACTGTTGGCTGCGACGTAAATGCGGGGAATGCCCTCGGCCCGAGCCAACTCAGACGCCCTGAGGAACAGCTCATCCTCTTGAGGACCAAATGAGCCGATCATGTAAGTGATGTCATTGCAGATGACAATGATATCTCTGCCCTCTGGGTACTCTGGAGTCTTCATCGTCATTTTGAAGGCAACCATTCCCACATCATTGTCTCCAGGCAGGCGGTTCATCTGCACCAGTTGACCTTGAGGGTCCAGAACCAGCTCGGTGCACATCAGCACGTCTTTAGGGTATTTGTCCCCTGGACCCCACAGTTTAAACAGTGCCTGTCTGAACATCTCAGGGAAGTCATAGACGTATGTAGTGCCGAGAGATTGAGCCTGGAAGCGTTTGGCCTGCAGCAGGTCTTTGGTCACATAGGGAGTGCTGATCAGCATGCCATGCAGAGGACCCTGCTTATCTCCATATGACTGGAACATGATCTGTCCAGAAATTGGGTTGGTGACCTCCTTGTACAGACTGATGTCCAAGTAATAGCCAGACTCATTAGTGAGAAACAGACGGACAGGAATGGCATTCCCGGTTGGTGTCAGACGGATGTTGATCTTCAGCTCAGCCTGCAGGACCCGCAGCTTCCAAAGACGGCTGCCGTAGCGCATCACCATGGAGCGAACAGACTCCTCTATTTTAGAAGGGTTCATAATGACAGTGGGGACGAAGTTGAGGAAAATGTGGTTGCAGTCTGTGCGGACACCGGTGTTACTGAAGGCCACCTCCAACTCATCCATGGCCTCCAACAGGAGACGTTCTCCCTCATTTTGAAGGTATTCAAAGGAGGCTTCCTTTGTAATGAGATCAGAGTGTCGGATAATAGCTCGTATAAAGAAGCGATAGTCTGTGACTTCAGCTCCCTCCTGAACACGAGCAGCACCAAGGTACAGCTGCATCTTGTGGTTGGCACAGGGAACGGCTGTCAGGTCAAAGTTCCTCATGCGGTTGAGCTCCAGCTGAAACGCTAAAGCCGGCTCCAGGTTACGATAAATACGATCCTCCTGGAACCCATCTCTAGCTCTGAAAGTGAAGAACTTGGGGAATTCTCTCTTCTGTGCAATCAAAAATGTGATTCGCCTGATTCCATATTCAAAGAGGACAGCTCGCTTTGAGTGGGCGAAGGCAGTGAAGGCTGTAACCAGGGCATCATCATCTTCTGTGTCTGCTGTTTTTATGGACACGTTAATGATGTGGATCGGGTTTTCCCTCGTGTTCTTGAAGTCCTCCTCTTCAAACAGGCTGGAGCAGGATTCTGAGAACGGAGCACTCTCCAAGAGTGGTTCTGCAAAGCTGGTGAGAACTTCATCAAAATTCCTTTTGAAGTCGTCGAAACACTGGAAAGCCACCATGGCGCCCATGCGCTGGCAAGGGGGAGAAAAGGCTCCTTCCAGAAAGAGTTCACTGCTCTGGCGCCTCATTTTAAAGTGGCTTGATCCACTCACTGGCACAGGAACCCTGAAAAAGAGACACACAGGCAGTAAGAGCAAGACTGAACCACTGAATGATCACATTGAGCGGTTTGCTTAAGCAAAACAAAAACAGCCAAGAGTACAAATATGCATACAGAGAGCAATTAAAGCAAGTCTGGATGTTCTTATGTATTTGACAAAAAGCCAGAACGATCACCCTCATGTCCAATTATAACTACACGCAGTTGATGGGCATATTTCATGTATTGAATTAAAAGGGATTATGAATTGTTGTTATCCAAGCAATTGTTACTATATCTGATTGGGAGTTGCAAATCAAGAATCACAGTCTGAAGCTTTAGAGTACGGGCTCTCCATTGGGCAGCAGTCAACACAATCTCAGGCTTGAAGTTTCTAAGAGGAACATGGAACAAATAAAAGATATGAACCTCTCAAAAAGCTCAGTATTGCTAAGCAACAAAAAAACATACGAAGAAGCTCCGCTAAAAAGTGAATTAAGAAGGACTTGTTAGAGGAGTCCTTAAGCAAATGTATCTGGTTCCAGAGGACAGGAGGAAAGAAGCAACACAGCACATTCTGCATGCATTGCTGTGAGACATACGCACGATCAGGTGTTTTGGCTGGTTGGGAGGAACTTTACTCTACCTGTTGAGAGTAGGGTTGCTCCCTCTGT

1. Hormone-sensitive lipase (*HSL*)

TGGGAGAATATATTTATTTAGATAAAAATCAACAAAGGAAGAGACCAGCCTACTCGTGGCGAATCAGACACCACTTTGTAGTATGAAGCCTGCAATGAAAACGCAGTCAACTCAATCTGTAATCAATCCGATTAACAAATCTGAAAGTAAAAATATCTACATGAATCATATATTGTTTAATATACATTATTTTTGTATTCACAGGTTTGCACTTGTGGTGTTTTGAATAAAAGAAAAAGAAAACAAAGTAAAAAGAAACAAGTAAACTTAAAACATCTTTGTTTTCCAGTCTATGACTTGTTTTTGTTTATGTATTTTTAAATTTTTTTAAAGGCTTATCATATTCTACATCAGAATTACATTTTACAGTGCGGAGCAATTTGCATATGGAGTTCAGACAGGCTATGGCACTGACTAGCACCTGCATTTTTTAATGATACCGGTTAACGGCAAACACTAAACGGCAAGAACAGCACATCTGTTACAAGACTGGCTGCAGTCTTTGGTTCTGCTTCAAAACTAACAATACATTTAGGATACAAGTGACTGATTAAATGAGCATTTACCATTAGTAAACAGTGGTTGAGGTCAGTCACAGACGCATTGATGTAACAGAATGGTGTAAAACAAGTGTTGTGGCTTATATGCTGGTTCCACTACCAAATCCCACCTCAAGGTGATTCAAACGCATTGCACGACAATAAATGGCATCACAGCATTTCTGACTGAGGAAACAGACACTGTGGAAAGATGGTGTGACGCTGGTGTGTAACTGCATAGCTTGACTTTAAATGTGGAGTCAGTTAGCTCATAATTTTAGCAGCATTGTACTTTGCATTGCACTTCAACGCAATCAGCCTGCGGCAACGAAAACCTCAAGTGAGAAGAGCTTTTGAAGACCAAAACTGTGGAGGTATGCTGTACAACCTGATAATTAAACAACCAAAAAGACAAACGAAACAAAAAACAACATTACACTGATCTAATATAACTAGTTTGCAATCACAAGGACATTTACAGAAAAAAGTTAGTCTGTGGCTCATCAACAAATAGACAGATATTAGCCCTAGATAGCAACACTAATTGAAACAATACTGAATCATGCATTTCAGCTCTCTTCTGCAGGAGGCATATACAGTATATGCTTCGGCAAAACCCTAAAAATGCCAGGAATTTGGAAAACTAAAAAAAAAAGCTAACACTGCAAACGTATTTGTCCATAACAGCAGATGGTGTCTCTGGTACATCACCTTCAGTCAGGTTAGCCCGACGCACGAGTGGTCATCAAGGGTAAAGAGGCATATGGAATTCAGTAAACAGCAAGTGGGTGAATTTGACACATTTTTCAAGTTTTCTAACGGCTAACATACTAAAGTTTATACAACAAAAGTGCCGACAACCAATCAACCCTGCTGGGCTTCATGGGCGGGGGGGTGTTTTCAGGATTGATTTAGAAGTTCAGTCGATTTATACCACAGACAAAATATGTGCCAAAGCGGCTTGAGGTTGCTCTCAACAGCAGATGATTAACACCACGAAGAGTCTCGTGACTGTAAAGTGCCGTTCCTGTCTTTCTGTGAACAGAACACACGACGCAAACAAGGAGGACGCGCTCACGTCTGAGCTCAGGATTCTCACACACCAATGAGAGACTAGTGTGATCATGTGAATGTGTACATGCCGCCGCCGCTTGAGCCTCACGTACAGGCACCCAAAAAGAACAAACAACATGTACACAAGCTTTAAATACACGAACAGATTGACAAACTACTGCGAAAGTGCCGAGTGAGCATAAACAGCGCCTGGAAAAGCAAGACCAAACTTCGGGCCAGTATTTCAGAGTAGATGAGAGTATTACTTTCTTTCTTTTCACACCCCTGGTTCTCTCCATCAATCTGACTAAAAATATTGCAGTAAGTACTCCGAGCTGACAGAACTGAGCGTGGTCTAATCCCGTAGCATGACTTTGCTGCTTAAAATCCTGCTCTTCTACGGGGAGAGGATTTTGCACTCAGAGCTTCAGCTAAATCCTATTCATCACCCAGCATCCTTCTCTACAGACCTGGCAACCTTAGCTCTGTTACCAGGCAAGGGCAGGACAGTGCCAGCATTGTTGGCATCTATATTTAGTTGGACCTGAGAGGGACTGACCCTCCGAAGGACATTTTTTTTGTACTTCAAAGTGCTTTGGTGGAAACATGCTCAGGAGTGCCCTCAGGTAATTGAAAACAAAGTCAATCACAAAACGTGACGGTGGGGAGAAAAAAAACATCTGGTCCACCAGACACTTTTAGTTGGCCAAATACAAGTTCAGTTACTTTTACATGGAACCCCTAAAATCTCTTTTTCTGGTCAGAGTGCTCGAGTTTTGCCTAAAAGCATCCGATTTGAAGAAACCAGATCGTCAGGCAGGAAATCCCCTGACTGAAGAGAAGTGGTACTTTGACACAACGCATACTGTGCAGAGAAGTGCGCACAAACACCTGGCGGGGAACGGGGACAACCTCAAATCAGTGCATTGGAGTTTAGACTGTGTATCGATAGCTGAGGGCTTTTGGTGACCTTTTCTGTCTCACTTTAGTTCTGCAGAAGGAGAACGTTAACCAATTCTCTCCAGTATCGTAATAAAAACAATTTAAACATTTAGCGTCTTTCAGACGTCTGAGAGCAATGCTTGCTATTAAAAGGACTTATGAAGATGGGAGACACATTTTAAAGCAAATACTCTTCAGAAAATAATCCAGCTCAGTGCATCTGCACAAATTCCACTGGAGGTATTAATAAAATGCAATCTTTGCATTAGTTATATCAGCCCTACAAAAAGCAGCAAGCAGCGTCTAGACAGGGCACCGCGTCCCTGTGTAATATTCTACATATTCTACTTTATCCTATCAGTATGCATTGCTGATAGGAGGATTTGAGTTTAATTGGAAGGGCCAGGGATGTCGGAGCAGCTGTGCCAAAAAAGCAGAGGTAAATATTGCGTACATTCAAAAATGTGGAATAAATAGGAAAAGATTACAAACAGTATTGTGTACAGATCAACAAACAAACCTGTGATGATTGAAGGATTAAAATACAGCAATAGAAAAAAAGATATAAATAACTGAAGTATTTCTACTGCATAGGGTTAATAAGATGATTAAAAACTGCAACAATTTCCAGAAACAAGTAAATCAATTCATTCAGTTTAGAGCCGTTCAGTCGGTTCTGTTTTAGCGTTGTAATTTGATTTAGGAGAGTCAACAGAGTTACTACCCACAAAGTGAGCTGTATCGACTTGCAAACTGGCGCAATGGAGAACACAATATGGAGTTATTAAGTGTGATTTAGAGTCGCTCCCTAAAGGACAAGTGGCTATTTTGATGGGGATGGCCCCATAAGCATATAGTGTGCCTACCACTATCTGATTAGGGCTTCAAATGACTGCCCTGGCTGAGATGAATCACCCATTCAGGCTTCGTCTTAAGTATCTTATGCGCCGCTTTATGCCTAACTGCAACTCTATCAAGTATGTGTGGGCGAGTGCTGGTCTTTGATCTAAGCAACTTCGAAATTACCATTTTATTCGAAAACACAATTGTCTTTTGTGGGCGTTTTCATTTTGTCTGCCATGCGTCGGTGCATTATAGAGCACAAAGTGGCAAGAATTGTTAAGCGGAGGAAGTTGTATTTCAGTGCTCCTCTAATTTTTTTGTCTCTTAAAAACTAAGCATTCACCGAGACGGCTGCCCTCTCCACATCTGTTTTTAGGATACCTTCTCTCTCTATCTGCCTTGCACTGCTTACATTTGTCTGACTTTCATTTACTCGTTCTCCCTTCACTCCGCGGATCAACTGCAGTTTTTTTATCTAACCTTATTTACGGTCCAATACCAACAGCGTCAGTATTGTTCTGGGCTGCCACAGCAACTGAGCCCTCCCCATCAGCAATTTTAGTCCCCCTCCCGACAGCAAGCTCCCCTCCCTCAATGGGGCCGACGAAGAGAGAACCGGCTTCCCCGGAAGAGGCTGACACACTCCTATCGGTCCGTTCCAGCTTGCGGTGCTTGCGTGGCTCCGGGGGTGTGTCCTTCTGGGTGAAGACAGTACGAATTCTCTCCACGCAGACATTGGCAGCCTCCTTCGTCTCCCTGCAGAGCTGCGATAGGCTGAGGAAGCCGTGGGGGAGGTCGTCCACCACGCACAGAGTGACAGGCTGGTCTATGTTCCTCAAACGCTTGGCAAACATCACAGAGTCATCCAGCATGGGGTCCAATGCACAAGCCACTATGTGTACAGGTGGCATCCCTTTCAGCATGCTATCAGGAGCCAGCAGAGGAGAGCAGAACGGATCCTTGACCACGGGAGAGCTCTCCATCGTCATCTCAGCTAGCTGCTCTGAGCGCAGTGGCTCGAACCCCAGGGGGAACTCCCTAGGGTGCTCCAGCTCTGATCCCTCCTCTCCAGCAGGGGGTGGTATGGCCACAGAAGACAGGTCCCTGGACATGGAGGGATCTTCATCCTTGGAGAGGTAGAAATTCACATCTTCTGGAGTGCGCTCAGAGAGCAGCGGGGCACTGTGGGAAGTAGAGTTGTTCTGGGATCCCAAGTCCAGGAAAGTCTGGCTCTTCACGGATAATTTCCTGGTGGGGAACTCTGAGGTATCTGAGGCGATAGGGGGATCGGCGTGAGGAGAAGAGATGGACGCCTCTGAAATGCTCTTCCTCACTGTGTCTGTGGCTCCTCGTGGCCTCTCTTCAGCTGTGCTGGGGGAGGCTGAGGCTCTGTTGGAATCCAGCAGAGAGTGGATCCAGTTAGAAGCTCCCTGTCGGAAATCTCTGAGCAGCAGAGCAGTGTCTCTCCTCACCAGGCTCAGCGTGCTCACTTTCTCTACCTGCGCCTCCGTCTGCGGCTCATTGCCTCCATAGGCGTTGAGACACCTGGAGAGCACACTGAGCGGCAGCAGGGGATCCATAAGTGTTAGCAGACGGGAGGGCGACGCGTAGGCAGTCAGCAGGGTAGCCGGGTAGATTGCGACAAGGCCATCTGGCATTCGCACACCAAAGGCGGCAGCGCGCATAGATGTCGTCACACACAAGTTGCCTCCCGCACTATCACCAGCCAGACACACTTTTTCTCCGGTCCAGCCCAGTAGGTGGTGATTTCTCAGAGCCCAGCAGTAGGCGTAGAAACACTCCTCCAGGGCCCTTGGGAATGGGGCCTCAGGGGCCAGAGAGTAGTCCACTGACAGTATGGGGACACCAAGGTCCTGGGACCAGCTCTTCAGATAGGGCTCATGTGACTTGGAGGTCTGGGCCACAAATCCTCCCCCATGGAAGTGGATCAGGAGGCAGGGAGATGAGGGGAGGCGCTTGGTCTTCAGCCCCAGCGACAGAGAGATGGCGCCCCCCTCAGAGCGAGCAAGAGATAGCAGAGTCTCACTGTCCTGTCCTTCACGTAAGTCATATGAGATGAGCCTCATCTGAACTGGAGCAGTCCCGATGTGTGCTGATGGTGGAGCTATAGTTACAGATGCTCTGTGGTTGGCCGCCAGGGGAAGGTCAAAGGGCACGGGGGGCACAGAAAGAGCCCGGTTCACCTTAACTTGAGTGGATGTCATACTGGCAAGACTCGACAGGACTTCGGTCTCAGTGATGTTCCAGAAGCTCTTCCAGAAATGGACGTCCAGGTTCTGGGTGATGCGTTCAAATTCTGCCCCTCTCAACTCTGGGTCAATGGCGTACTTCCCTGAGGTAAAGAAGGAGCTGGCTGCGACACCTATTCCAGACTGATGGCGCTTGTAGTTTTCTCCAAAGGCCACAAGGCCAATAGCGATGGTCTGGAGACAGGGTCTAATGGCTGGACTGAACTGAAAGCCAAGGCAACGGCCATAGAAGCACCCCTTGTGCATTGATGAGTACTCCCGGACAAAGCTCTCACTGAGGCCATTTTCACCATGGAAAAAAAGGTTGCCATGGTTGTTGTCATGTAGCATGCGCTGAGCCAGGTAGAGCAGGGCGCGCAGCTGGCACAGAGCGTTGCAGTAGGCCTCCATCTCCCCCGCATTGTGTGCTAATCGAAAGAAGATGCTGCGGCGGTTGGCGGTGATGTAGCGTCCCTTCTGGATGATGTGGAGAAGGCAGCAGCGTACTACCTTGACCAGTGAGCGATAGCCATTGGCAGGTATGTGTGGGTCAAAGTCAAAATGATGGTAAACTGCAGCAAAGCCAGAGACAACAGGCTCCAGACTGCGACCATGCTCCTGGATCAGCTTCATGGTATCCACCAGCCGCCGGGCCGCATCACCCTGCGACCCTTTGGCACCTCCTGAGAAGAAGGTGGCATTCTCTTCACACACGTTGTACAGGGCCGCAAACACTGCCTTGGTGTCCATCACAGCATCTGAGATTAAGGCTCTGTCCTCGTCTGCCTCCTCTCTTCCACGAAAGCAATGAGAGCATGGAAAAGAAGAAAGAGAGAATGAGAGAACGAGAGGGATGGCAAAGAGGAAGGAGATCAAGCATTCCGATTGGTTGTTCTCCCTTTCACCGTCGGTCACTGCCTCATCTATACAATTGGATGTGGCTCTTCTC

1. Carnitine O-palmitoyltransferase 1 (*CPT1*)

CTCGGACATCACTCTACCAACTGCCACTGCGTGATTGCGACATTTCCGACAGCATATGAAGGCAGCATCAGCCTCATCCTGGAGGAGAGCATCTGTAGTTGCGGGTTCGCACAGGGCCGACACCACTTAGCCGTCGTGAGGAGTTACCCGTCCCGCTCCTTTGGACCCCTGGACAGCTAGTAAACAACAACAAAAGTCATCATGGCGGAAGCCCACCAGGCAGTGGCCTTCCAGTTCACGGTCACCCCAGATGGCATTGATCTTCATATGTCCCACCAGGCCCTCACTGAGATCTACCTCTCTGGCGTGCGCTCCTGGAAGAAGCGTATCATCGGGCTCAAGAACAGCGTGATAACGGGGGTATATCCTGCGAGTCCTTCCTCCTGGCTTTTTGTGGTCATAGCAATCCTGGCTACTATGTACACACGCTCCGACCCCTCCATGGGACTCATTGCCAAGATACAGGAGCACCTGCCAGTCAGCCAGTCCATGAGTTCCCAGTGCCAGACGTTGGTGTCGGCAGTGCTCTTCAGCACCATGCTATGGCTCTTGCTCATCTTCACCATGCGCATGTGCCTCAAGCAGCTTCTCTCCTACCACCGCTGGATGTTTGAGAAGCACGGCAAGATGTCCAACACCACCAAAGTCTGGGTGGTCAGTGTTGTCTCACACTTTCTTCTATTTGTTACTGTGAACTAACAATACCCTTCGCTTTCCTCAAGTCTTGTGATCTTTGGTATGCAGCCTGAGGCGCCACTGAAGGTGACACTTTCATATGCGGTTATTGCAAACACAATTAGTGCAGTGTATTTGTTTCTGTGGCAGTTTTTATGTCCTGTCATACTACAAAATGTTGTGTGTTTTATGCTGCAAGAAAACAACGTTGAATTATTCAGAAAATGCTTCCAGTAATATAAAGTGAAGAGATAAATAAGAGGGAAGTTGTCAATCGAAGTAAGTTGAACTACCAACCATCGAATAACCTTCATTTAATAATATATTTGTAGATCTGATTCTTATGATATATCATAGTCTCCAGAACTACTTGGTGCCCTAGTTATGTCCTCACTTTAATATTTTGAGTGTTTGTTTTTGAATGCCCGTGCTTCTATGCCAGGCGCTGGTGCGGATCTTCTCTGGCAGAAAGCCTCTGCTCTACAGCTACCAGGGTTCGTTGCCAAACTTGCCTGTGCCTGCCATCAAGGACACAGTCAAAAGGGTGAGGCGTCACATGAAGCAGAGGAAATGACAACACGCTGACAACGGACTGTGGAACAGATGGATTGCTTTCGCACGTTCATCATTTTCTGCCTCTGTTGCAACAAATTAATTTTGTTATTGTCTGAGTGTTCTGGCTGCAAGCTCGTTTAGAAATATAGAAAATGCATGCCATCTATCCTTCTAATCGGATATATTTTTGACAGATGAGAGGAGAGAGATTTCAGTATAATTAAATGTGGTTGTAATGGTAGCAGGCTTCCACGTCACCGATCGTGGCCTTTTATTGATGTTATTCATTGATTTACAAACCTTTTTCGATGTTTCTGTATTTAGTAGACTATGTAATCATGTCGGTCATTTTATATCTGCTGAATGATGCTTCTTCTTCTTCTTCTTCTTCTTCTTCTTCTTCTTTGCACATCTCTTTTGTTTAGCACTTGGAATCAGTGCGTCCGCTCATGGATGACACAGAGTATGAACGCATGACCAAGCTGTCAGCGGAGTTTGAGAGCAGCCTCGGTAAACGCCTGCAGTGGTACCTCAAACTCAAAGCTCTATGGGCCACCAACTACGTCAGTGACTGGTGGGAGGAATATGTCTACCTACGAGGACGGAGCCCAATAATGGTCAACAGTAACTATTACGGCATGGACTTCATGTATGTGACGCCCACACCCATCCAGGCGGCCAGGGCAGGCAACAGCCTTCACGCATACTTCCTATACCGCCGCAAACTCAACAAAGAAGAGCTTAAACCTACCCGTATACCCGGCACTATCATTCCTCTGTGTTCAGCTCAGTGTGAGAGGGTTTTCAACACCACACGCATTCCTGGAGAGGAGACCGGTAAAGAGACAGACATGCACATGCACACGCACATAGACACACGGTGCTGATATATGATGCCCAATATTGTCTGAACATGAGCAGGTAGCTTCAGCAAGTGAGCAGTGTCAATACGCCACTGCTTCGGAGATGATGAGGCATACGCAAGGTGTCTGAGCCCTCTGGAGTTTAAGCGTGGGAGAGAAAATTGTGTGAAAGACAGTGTGTGTTATGTGTGAGTTTGTACAGTCTAAAGAAGCAAGGTGCCGTGATTCACTGAGTCATAGTATGTCTGCTGCACAGGCCATCCTCTCATCTCTCATCTGTCTCCCTCTTGCCTTCTGGTCTCATCGCAGGTCTGTCTGGGTTTCTGGAAATGTTCTTTCTCTAACTAATGTCTATTTTCTTCTGCCCACACATTTTGTTTTCTCACCTTTTTCTTCCTCTCTTCCCTGGTTCTCTTTCTATTTCTTCCAAACACTCACGTGTGGGTTTTTCTATCCTTTGCATTTTCCCCTTTCTCTCCCTCACTTTCTTTTTGTCCTGGCCCGTCCTTCTTTATCGGGCTCAGTGGTTGTTGAGGTCGGCCATTCCTTGCTGTTCCTATCAATTTGAGCGGATGTTTGACACTTGTCGAATCCCTGGAACACTAACAGGTAGTATTTATATTTTTACACAACTGGCCCTTTTAGATCTCTAAATGTCAAACCAATATGATAACATAAATGTGCATAACATTTTTTACTGTGTATAATTAAGAATGTAAAATGACAATACATCCCTGTCTCATATATAGTAAAGGTTCCCTGTCCATTATGTTAATGAGCCAACATCTTGATTGAGTTGTATTGTGAGGTTGAAGAAGTTTCCCTGCTCATCTGAATTCAGTCGGTCAAACGTGTGCCAGCGTGAGCGTTTGCATATGGATCAGATCTTAATCACCCAGCTTTGTGGTCTTTACAAGATGGCAGAGTAGAGGCAGATCACGCCACGTCCTTCACATGTATCTCCACATAACATGCATCATGTTGTTTCTTTAGAATATTTATCTGCTGATTATCGTGTGCTTTCAACCAGTGAATGTCACAAAGACATGTTGCAGATGCAGCTGTTGTGTCCTGCAGTCCCAACAAAAAATGGATGAATCAAGAATGTTACAGGAACCCAGTAGGGCAGACAGTTTTGAATATACATAAACGCAAGGCACACACAAATGAAGTGCATCATCTCTGATGTATTTGTGTTTCTATGACATTTCTCTGACTCAATTAACAAAATATATGTTTTTGGTTTTCTTTAATGTGCGAAGACACTCTGCAACATTGGCAGGACAGTGACTACATCGCGGTATACCACAAGGGTCGCTACTTTCGTCTCAGGATGTACCATGCAGGCAGACTCCTCACACCCAGGGAGATTGAATCCCAAATTCAGAAGATCCTTGATGACCCGTCACTTCCTTCCAAAGGAGAGGCCAAACTGGGGGCCCTGACCGCTGGAGACAGAATTCCATGGGCTAAAGCCAGGATGAAGTATTTCAGCAGTGGGGTCAATAAACGGTCTCTGGACTACATTGAGAAAGCGGCCTTCTTTGTGACCCTGGATGATGATGAACAGGGCGGGATGGCAGATGACCCGACTAGATTAGATTCATACGCCAAGTCCTTGTTGCATGGGAAATGTTTTGACAGGTGGTTTGACAAGTCCTTCACAGTTGTTTACTTCAAGAATGGAAAAATGGGCTTAAATGGAGAACACTCCTGGGCTGATGCACCGGTGTTATCACACGCTTGGCAGTACGTCTTGTCCACTGACTGTTTCCAGCTCGGTTACAACGCAGAGGGTCACTGCAAAGGAGACGTGGATTCATCACTACCACGACCACAGAAGCTGAACTGGGAAATCCCTCCAGAATGTGAGGAGCAGATCTCCGGCTCTCTGGCGGTGGCCCAGGCCCTCGCTGACGATGTGGATGTCCACGTTTTTGCCTTTGAAGAATTTGGCAAAGGAAGAATCAAAAAGTGTCGAGTCAGTCCAGATTCCTTCATTCAGTTGGCTCTTCAGTTGGCCCACTACAGGGACCAGGGGAGATTCTGTTTGACGTACGAAGCCTCCATGACCCGTCTGTTCAAGGAGGGCAGGACCGAGACTGTTCGCTCCTGTACCAACGAGAGCAGTGCCTTCGTCCGAGCGCTGGAAGGTGGAGAGGCAGCAGATGTGTGCAGGCGCTTGTTCCAGGAAGCATCGGAAAAGCACCAGTATCTCTACCGCATGGCTATGACTGGATCTGGCATCGACAGACACCTCTTTTGTCTCTACGTGGTATCTAAATACCTCAGAGTGGAGTCTCCTTTCTTGAAAGAGGTGCTATCTGAGCCCTGGCGGCTGTCCACGAGTCAGACTCCATTGCAAGTGGAGATGTTTGACATCAAAAACTACCCAGAGTACGTCTCCTGTGGAGGGGGCTTTGGACCGGTGGCTGATGACGGTTACGGGGTGTCCTACTGCGCTTTGGGAGAAAACGTGATCAACTTCCACGTCACATGCAAGCACTCATGTCCGGACACTGGTGCCAATAAGTTTGGTGCTCAGATTAGAAAGGCTCTGCGTGACCTGCTTCAGCTGCTGTGCCCCAACCAAACAGAGTTCAAGAAGACAGAAGTGAAGCGGCCAGAGTTCAAGAAAGACCTGTAGGCGGTCACTCAGGGGTCAAGGAGGCGGAAGGGAAGTTGTGTAAGCGTTTTAGCAAGCCAGATGATATTCAAGGGGCAGCTCCATTGGAAAACGAATAGAATGGAGGTGATGTTTCTACTGATATGGATCTGAATTTGTGTGTGTGTGTATTAGCTGTGTGAGAAGGAAAAAGGTCCACAGATTAACAGATCAAGAAGTGGGATTCAAGTACAGTAGGATTTATTGGTTTAGTTTTAACCGACAAACCATTGGAAAAATATAAGTCATTATCATGACATGTTATAGATTATTACTAAAAGGCCAGTATGAATTGCACCACTCATAGTTAGGGTAGAAATTCCTAATACTTCACCTTATTACAGGGATTCATTTTATATATCTTCAAATGCAGTACTTTTTGACACCATTAAATGTGCCACACTTTTTGTGCCACTGGTTTTCTGTTGGTGACTTTATTTGGAGAACGTGTTTGGAAGTGTGTGACGCATCTGGAGTCTCGGCACATTAGCACAGAGAGAAAACATGGAGGGCTGTTGAGGTGATTTCATTTTCTACCATCTGAATGCGACATTGCTGTAACAGTGACACCACACATTTTGAAATATTGGAAGTCTGGGACTTCTCATTGTTTACTCGTTATGTATAAATCTGCTTTCTACAAAAACCACATGATTGTAATACTGTGCTGAGAGTGCTGGGCTGTTTATACATGTAGTGAAATGACATGGCTCTTTAAATTCATAGGTTGGCATTACTGTTTGACTAATATTATATTCAGTGCCAGCTCACCCAGGATATAACTTTTATGTTTTCAAACATATTCAAAATGTACATACAAATAATGTAGGTCAATTTGACTTTCAGTGGTCAGTAGGCAATTCAAACTTATGAAGTAAAAAGCTGTACTGTAATGAACTCCAGATATTTCACTGCCATCTATGAATCTGTTTTGTAATTATTTTCATCCCTGTATTATTAAACAAGCACCATGACAATGCAAGCTATATTTATGAATACCATATGCAGGTATTTGTTTAGTCGTGAGCCGAGCTGCAGATCAGATGTGTGTTTCATGTGGAGTGAAGCCATCGGATTCAAAGAAAGTTGTGTACCAGACACACAGATTCCTCAGTTATTCTTCATCAATTATTGCCTCTTTTTTTTCGGTTGAGCTTGTCTCGTAGTATTACTATGGTCAGCCTTTTGTAATGCAATATTACCGCAGAACATAAAGGAATTCTTAATGAAAACAGCTTTAAGTGTTTCTTTCTGTTGAGGAGAAGAGGACTCCACTGATTTATTAGCATTGCTGGACTTATGATGGCCCGCTTAAAAAAAAAAAGAAGATCTGAATTGATGCAGCAGAACCAGCAATATCTCTTTCTTTCTACTCCCTTCTCAAAACCTGGTGCCTACATTACCCAGAATGCAGCTGTACAGTGGACGTCACTTCAATATTTTAGATTTGGCGCAGCGCCCTCTAGAGCCA

1. Peroxisome proliferator-activated receptor gamma (*PPAR-γ*)

CCTCCCCCAACAAATCTGACGAATCAGACTTAGTCGACACATTGCATGGACGAAAAATAATTTTATAGCAAAAATGATAACTCAACCACTTTTTAAGAATTTAAAATAAAACAAGGCTGAAAAGTGTGTTTGACAGACAATTTCATCCATACAAAAATAATACATATGAATTTATACTATACATTATACATGTGTAATATATATATATTTTTTAGATGGCATTTATTCCACACAAAATATGAGTCAAACAGCAATCCTTAAAGGCACAATATGGCGTGTACGGTTTCACCGTCATTGTAATGTGCCTTACGTTGGCTTCACACTTTCTCACAGTTTCTGCAGGTGGCCGAGACTAAAGTACGTTACAGGCAATAATCTGATTTCTGTCTTTGTATAACGCTGATTAAAAAAAAAGATAGCACCTTCACCCTGATGCCAAAACAAAAAAATTACATGTGCATTGTTTTGTGTGCGCATGGGTGAGCATTCATGCATTTGTGTGCGTTCACAGAGGTATTGAAGCACGTACACACTGCTGCTGCCTATGACGGTTGTAACGCAACGTTTGGATAAAGCTGCACATGAACAACGCATACGCTACTGTGTAACACGTGTTGTGAACATATTCACAGTGATTTCTCCTGGAGGCACTTGAAGCAAGCTTCGCCATTTTAAGCTTCAGCTTGCAAAAAAAAGAAAAGAAAGTCCCAACTCTTTCAGTTAGTCACAGACTCTTGTCCGGATTGGTGGTAGTTTCGTATTTTAGTTGGCAGCCCACTCATTCACAAAGTGTCCGGGCTTAGCAGTGGCTAAATGACTGTGCTGCAAGTTATTCACATGCTGCTGTCTAGACAAGATTGAACACTCAGATTCTGTCTTTCTGTCCTTGATTTCTCTTTTCCTGTTCTTCCTCATCTCTTTCTCAATTTCAGTCAGCCTGTCTGGCTACCTCCCTTTATTTCAACACACCAGCAGCACAAAGTACTTTATCATTATGATTTCACAAGTCATGGTTTTAATAATAAAATCTCTTTCATTCCACTTCCTCTACTTGTCTTTTTTTCTCCTGTTGAATTCTAATACAAGTCCTTCATGATCTCCTGCAGTAATGGATGTAAGCACATGTCCACCTCCGTCTTCTTCAGCAGCTGGATGAGGTGGACGTGGTCGGTTACGATCTGACGCAGGTCGGTCATTTTCTGAAGCAGCTTGGCGAACAGTTGCAGAGAGTCTGGGTGGTTGAGCTTAAGCTGCAGCTCAAGTGAATGGAGCACCGTCTCCTGAAGCTGCTCGATGGGCTTCACGTTCAGCAGGCCCGGGCGGTCTGGACGGGTCACAGAGACAAGGACAAATCAGTGACATTTCCGGGCCTACGTTAAGTATCTCTTCGGTCAACACATCTGTTGGTTTAAACACACTTAATTTAGCTGTCCCTTATTACCCAACAAAGCTTTATTATTATTTACAATTGCATTCTTATCTTTATTTCAAGCTATAATCTTAGTTGTTTACGTTAATAATTGACTTAAATGTCTAATGTTTCACCCTTATAAAGGTTCAAGGCCAGCATTTTATCAAGTGGTCACACGCTAACCAGCAATATTAAACCACAGTTTGGGTTTCTGAGGGAGGTTGGTTTGGTTTGCTTTTACATAGGCATGAAAACGACGACTATCTACTAACTATCAAGACAATAGAGGGATGACAGTACAAACACTTTTATCTCTTTAATATGAATCATCAATAACAGTGGAAATCCTTGAAATACTTCAAATCACATATGATGTATTTACTATTTCTTGCTCCCACGTGAAGCCTCTGTGCAAACTGATAAATAGGTCAAGGCTGGCCATAGATAGTGTGTATAATGCAGTGGGTTCTTAACTTAGACTGTAAACAGCAGACAGCTTACAACACTTAACATTCACAGAGAACTAAATTGTCCTGTTGCCACATGTCTGCCAGAAACGACTACACGATCATAAAATAAATTATTGCTCCCAGTTACTTGTTTAAAATAATTATACATATACTATTCTGCACTATAAGGCTGAGTGTCCATATAGCGTTTTTATAGCTCGGTAGCATTCCTCCACAGTAAAGACACCAAAAGAGTTTTTTTATGCTGTCTTTGATGTACAAAACGTCGAAGCGCACTGAAGAAAAAAAAGCTTGGTGCAGCGCTTTAGTTTCAGAAGTTTTCTCCTCAATGGGAACAATTGAAAAAAGACTGTAAGGACTTTGAAAAGAAATGTTAGTCATTTTGTGAAATACTATTTGCTCTCGTCAGGAGCAGGTTGATACCACATTAGTCTGTTCAGGATGAAAGATGTAACATTAAGACTTAGTATAAAGACTCTGAAGCTAATACATCACCGAAGTTAGCTCTCTCCTGGCTGCAGCTTCATATTAACAGACAGACTGGAATCGAATTAATCCTTAAATCCGATTAAGACGAGGCCACTATGATCTATAACATATCCTGTGTGTAAAAAAACCCAAAAGACTCACCCCCGCTGAGGATAATGACAGCCAGAAACAGTGCCATGTCGCTGTCGTCCAGCTCCAGCGTGTTGAATTTGACTGAGAACTCAAACTTTGGTTCCAACATTTGACTGAAAGGTTTCCTGAGACTCTTGAGGAACTCCCGCGTCATGAAGATCTGTCCGTAGGAGATCAAGGTCCCGTCTTTGTTCATCAGAGGCGACATCATGATTATTAGGACCTCGATCACGCCGTACTTCAGCAAAGTCACCTGATCATTGAGATCCAGATCGATGAATCCTGGGATGCTCTTGGCGAACTCTGTCACTTCTCTTACTGCTTCGGCCGAACGTGATTGACAGCTGTGGAAGAAACGCAGCTCCAAAGTGTCCGATGGAATCTGTCCGCTGATGCTCATTCCCATTCCCAGAAAGCCGTACTCTGAGCCCTGGTGAGCTCCTATGAGTCCTCCGAGTCCTGCTGTTAGGGCGGATGACTGCTGCTGTTGCTCTTGGATGGGTATCTGCTTACAATTAATAAACTGCTCTCCTTCCATTAGAGACTTCATGTCATGAATGACAAAAGGCACGTTGTCTCCGGTCTTCCCAGAGAGGATGGCCCTGGCCTTGACCTTGGTGAGGGGGAAGTATTTCAGATAGGCCTCATACAGATGCCGGGCCAGAGCCCTCAGATCTGCTGCTTCCGGGTGCATCTGCACCACGTCCGATGAGAACTCAGCCAGAAGTTTCTCCTTCTCCGCCTGGGGCATTCGGCCAAAACGAATGGCGTTGTGTGACATGCCGACGTTGAGGCACTTCTGGAAGCGGCAGTATTGGCATTTGTTGCGTGACTTCTTGTGAATGCGACAGTGGAGATCACAGTGATCGTACACCAACTTTAACCTGATTGTGCGTCTGAAGAAACCCTTACAGCCCTCACAGGCATGGACGCCATAGTGAAACCCTGAAGCTTTGTCTCCACACACACGGCACTCGATGTTTAGCGATGCTGGCGTCGTATCGTCCTGGAGCTTGGAGAACACGGGATTGTCAGAGTACTGTGGAGGTGACTCCGGCTCCAGCTTGATCAAATTGTGCTTGTCTGGCTCCGTTCTGTAGCTGTGCATGTTTGTGTAGTCCATGTTGGTCAGTTGTTCTTCGTTGTGCGACGGACTGGGGTCGTAGGCCACGCCGACAGAGGAGATGGAGGATACAAGCGGGGGAGACAGGGAGGACTGGATGGAGGAGGAAGAGGAGATGGAGGCGTAGTCTAATGTGGACAAATGCTTCATGTCGAGGGAGTGGGAGCTGTCGTTCAGCTCTGACAGCTCCACGGTGTTCAGACTGAACCCGACGGGCCAAGCTAGGAGCTGCTGGGTGTCCACCATGTCTACTGAGTAGTTGTGACCGCTTTGATGGTTAAGCTCTCTGCCTGGTGTTTGCATGTCAAAGTCACACTCACAGTGGCCCACAGACTCACTGTGGTGAACACCTGACTGCGTGCCAGTTGTCAGTCCTGTGACCGCACCC

1. Glucose-6-phosphate1-dehydrogenase (*G6PD*)

CTGTACTTGTTTATTAAACCCATTTAGCAGAAATCATGTAAACTTTAAAGCAGCCTTTTTGGCTCGTTAACTCTTCAAATGAAGCGAATTGCAAGATTGTTTTATTTGAAGCGTTCATAGAGGGTTGGAGAGGTGACATTATCCGGTATCCATTATCCATCTTCTACAGCATAATCTAGTTCCCAAGAAAAGAAGTTGCATGTCCAGCTCGTGTCCCAAGAGTCCTCTGGGCCTCTCTCCTTCTGCTCACCTCGCAGACGGAACAGGGGACCTCATCCTGGTGTGGGACACTCACCCGCTGGCATTCCTCAAGTTCCTCGTTTGTGGAGGTGCACCGCGTGAAGGCCGTCCGTTTCTCTCTCCCCCCCAACAAAGAGGATGAAGCATATGAAGAGATCAGAGGTGGGATAGATATAGAAGAGGGGGACTACATTGAGACTGTAAGCAGAAGTGGATCTCAGCAGCACCTGGCAGAGAGAGGCACAGGACGAGAAATGACAAATGATCAGAAAACAACGACCCCCTTCTTGTGTGGTCTGTGCTGCGGTAAAGCTCCTGCTGTGTCAGTGTGGAACTGTGACGGAGAGATTCTGCCTTCCACAGAGATCCTATCCAGGATACATGGCCAGCTGGTGCGTCTGTATGCCAGGGGTATCGAGGATGGAGCAGCCATACACAACTGCAGTGAAGAAAATGACAAGTGTGAAAGAGGATACACCCGACACATATAGTCTCACCTACATTTTGAGTGATGAGGGAAAGGTGACAATGGACATGTTAGGCAAGGCTAGTTTGTGAATTTACTGAAACAGCCTTGCAGTCTGTCATGGCCAAAGAGTTCCTAATATTTCAGGTACTTTTAAATACTTTCTCATTGCAATAATAAAACAAGTATTTATTCCAAAATGAGTGTGGGTTTGTTGAACTCTTCTACTTAAAACACTATTAAAAGTCTTGCTAAATGATGACTTATTTGTAGTGCAGATATTTCTGATATACAGTGTTTGTATGCAATTTTTTTGGTCTGGGAATTTTATTTATTTTTACTTAAGCTTCCACAGAATAAAACAACAGGTAATGTCAACAGAAGTTTAAAGACAAAAAACACCTTAAGTCAATAAAAAGACTGACCCGACTCATTTTGTGCTTTATTGTTGTCAACACTAGAGTACATTCCAAACGTAGTAAGAGCAATAACAGCTTGCACATACATAATTGTGCAGTAGGAACTATTTTTTCTACTGTGTTCCAAAACTATTTTTAGTGTCAACATCCCTTACCGTAATGAAGGATCATAATATTATTATGGCATATAGTAATTGTGCAGTACAAAATAAAATAACACATGAAAGAACAAATGAAATTGTTCCAGAGGAGAAGTTATGTCTGTCTTTGCTGCACTCCCCATGGCCCTCCGAGTACTGGCAGGATGACCAAATGGTCTGTTATTGGTCAGAGTGTGTATGTATTTGTGTGTTTGTAGTTTGAAGGTCGCCAGCTAGGCCATGTACCTGATAGGGACAGACATAGATGAACAGAAATATCAGTCTGCTGCAGGCAGTAGAAAGAAATCAAATTTAAAAGTAAATCTAAGTTGTAATGTTAAAACTCATTCATGCACTGTGACAGATTCAACATTACAAATATAATTAACAAGCATTTGAAATGAGCTTAAAGCACCCACCTGGAAGTTATTCTTACCTGACCCAGGGCATTTACAGAAAGCCTTTATTCAGAGAGTCGCCTGTACAAACCAGACCAACAAGCCCTCCTGGTGTAAATGTTATGTGGCGTTTAGTGTCATCATGGTGTAGTGATGACTGTAAACATGGGCCTGCTTATAGTAGAGAACAGGTTTATTTATATTCTTAACATTTAAATTACCCTAAGAGTAAATATTGATCCTTCATCCATAAATTATAAGTTAAGAAATTCATGTACAAGTCTCATATGGCCAATTCTGTTAAAATGGGAAGAAATGCAGACAGAAGACTGCATGATTTTTGATTTACTGTAAGTTTAAAATGTACAGTTGAACACTCAAATCAGGCTACTGCAGTTATTGTTATTTCCACATTCATTCTTGTGTGATCAGAGATCAAAGGCAGTGGTATTTTTTCTACATGCAGACAAAATCAGACAACTTACAGCAGTGAAGGATTGCAGTAAACTTTATTGTTTGTAAACTTGTCTATTGTGTGTATGTGTGTGTGTGTGTGTGTGTTCATGAATGTGTGTCTGAATCATGTCGTGTGGGGCTTCACCCACTTGTATGTTCCCTCATAGCGGAATCCGACCCTCTTCACAAGGTCATCCGCCTCGTTTGGACCACGACTTCCATATGTGTAAGAAATTGGGTGTGTCTTCTCTTCCTCTATTTGGTGGAGGAAGGGGGTGAAGATCCTCCAGGCCTCTCGTAACTCATCACTGCGAACAAAGTGCATCTGATTTCCACAGAAGACATCCAGTATCAGCCTCTCGTAAGCATCTGGGAGCTTCACGTTCTTGTATCTGCTCCTGTAGGTGAGATCCAGCTCCGTTTCCTCAGGGCTGAAGTAAACTCCAGGCCTCTTGGTCATCATCTTCAGGTAAATGGCTTCATCCGGCTGCACCCGCACCACCAGCTCGTTCCTCTGACAGCGGTCACCAAATATGTCTCCTGGTACATCAGTGAACTGCAGGCGCACTTCTGCCTTCTGTTCATTCAAAGCTTTACCACAGCGGAGAATGAAAGGTACGCCATCCCATCTTTCATTCTGGACATAAAGCACAGCCGTGGCAAAAGTCGGTGTGCAGGAGCCTTCTGGTATGGTTGGGTCATCAAGGTAACCCAGCTTGGAGTGGCCCTCACCCTTAGGGTCCCCCACATACTGGCCAAGCACCACATCTGACCCAGCAACAGGAGCTATACACTTCAACACCTTCACCTTCTCATCCCTCACATCATCTGGGCTGGTGGAAGCAGGTTTCTCCATGGCGACCAAACAGAGCATCTGGAGGAGATGGTTCTGCATGACATCTCGAATGATACCAAAGTCATCAAAGTATCCTCCGCGGCCCTGAGTGCCAAAAGGCTCTTTGAAGGTGAGGACCACACAGGCCACACTGTTCCGGTTCCATATGGGTCCAAAGATGCGATTTCCAAACCTGAGCACCATGAGGTTCTGCACCATCTCTTTGCCCAGGTAGTGATCTATGCGGTAGATCTGGTCCTCTGTGAACAGAGAGGACAGGTGGGCTGACAGCTCCTCTGAGCTCCGAAGGTCACGGCCGAAGGGCTTCTCAACAATGACCCTGCTCCAGCCTTTGCGGCTCATGCAGTGGGTTCTGATGTTTGTGCTGACATGCTGGTAGACGGTGGGTGGCAGGGCCAGATAAAAGAGTCTGTTGGCGTCAGCTCCCCCGGGCAGAGACGACAGATGAGAGTCAAGTTGAGTGAAAGAGCAGTTGTCGTCATACTTGCCGCTCAGATAGGAGTTCTTACGGAAGAAGGCTGAGAGACACTCACTGTCTTCATCAGTGACCTTCATATGAGGCAGACATGCTGACTTGATGTCCTCCACCGTCAGCTTAGACCGGGCAAAACCAACAAAGTAGGTGTCATCTGGGAGCAGGCCATCTCTGAATAACCACCATAAAGTTGGATAGATCTTCTTTTTAGCAAGATCCCCAGAGGCTCCCAGGATGATGAATATGTGCGTGCTGGAATGACTGAACTGCTCCTCTCCATAGAGCTCCCTCCTGAGCTGTCCAAACACCTCAGAGCGAGTCAAAGGCTCAGAACGAGTCGAAGGCTCAGAGCTCATCTTCTGGCCCGTCTGCTCTCAGTCTGTCCGAACTGTGGCAATTCTCAT

1. *β-actin*

TCTCCCGTCCCGTCCCCAGCCAACCTCCCATCCCCGTCTCGCGTCGCAGCGGCAGTTCCACTCCCTCGCCATGGGCAAGATTAAGATCGGAATCAACGGTTTCGGAAGGATCGGGAGGCTCGTCGCCAGGGTCGCCCTCCAGAGCGACGATGTCGAGCTCGTCGCCGTCAACGACCCCTTCATCACCACCGAGTACATGACCTACATGTTCAAGTACGACACCGTTCACGGCCACTGGAAGCACAGCGACATCAAGCTCAAGGACGACAAGACTCTGCTCTTTGGCGAGAAGCCAGTTACCGTCTTCGGCGTCAGGAACCCTGAGGAGATCCCGTGGGGTGAGGCTGGTGCCGATTACGTTGTGGAGTCCACCGGTGTCTTCACTGACAAGGACAAGGCCGCTGCTCACTTGAAGGGTGGTGCCAAGAAGGTGGTCATCTCTGCCCCAAGCAAAGATGCCCCTATGTTTGTGGTTGGTGTCAATGAGGACAAGTACACCTCAGACGTTAACATTGTCTCAAATGCTAGCTGCACCACTAACTGCCTTGCTCCCCTAGCTAAGATCATTAATGACAACTTCGGTATTATTGAGGGTTTGATGACCACTGTTCATGCCATCACTGCCACACAGAAGACCGTTGATGGTCCCTCGAGCAAGGACTGGAGAGGTGGCAGGGCCGCAAGCTTTAACATCATTCCCAGCAGCACTGGTGCTGCCAAGGCTGTTGGTAAGGTTCTTCCTGAGCTGAATGGCAAGCTTACCGGTATGTCTTTCCGGGTTCCCACTGTGGATGTGTCAGTTGTTGATCTCACTGTTAGAACCGAGAAGGCTGCATCATATGATGACATCAAGAAGGCTATCAAGGCTGCATCCGAGGGAAAACTCAAGGGGATCATGGGTTACGTCGAGGAGGATTTGGTCTCCACTGATTTTGTCGGTGACAGCAGGTCGAGCATCTTTGATGCCAAGGCTGGAATTGCTCTGAACGACCACTTCGTCAAGCTTGTCTCGTGGTATGACAACGAGTGGGGTTACAGCAACCGCGTTGTCGACCTGATCCGCCACATGGCCAAGACTCAGTAGAGCGTTCTGCTTTCGTTTGTGGCGCCTGGTCAGAGGTGTCCCTGCTTTGTCTATGCGAAGAATAAATGTGGATGGTGTCGCCAGACGACGCCTGGTTCATGTTAGTTGGACATGAGTTACCTTCTGTTTTCCGC
